# Adipocyte‐Derived KIF13B Aggravates Obesity‐Associated Metabolic Dysfunction via LRP1/PPARγ Signaling

**DOI:** 10.1002/mco2.71003

**Published:** 2026-09-16

**Authors:** Guolin Miao, Zhaoling Li, Lianxin Zhang, Yitong Xu, Ziwei Wang, Zixun Wang, Aiden Xian, Liwen Zheng, Jingxuan Chen, Yufei Han, Chunhua Shan, Kaikai Lu, Ming Tao, Ling Zhang, Weiguang Zhang, Wei Huang, Yuhui Wang, Rui Wei, Erdan Dong, Xunde Xian

**Affiliations:** ^1^ Institute of Cardiovascular Sciences, State Key Laboratory of Vascular Homeostasis and Remodeling, School of Basic Medical Sciences Peking University Beijing China; ^2^ Department of Cardiology and Institute of Vascular Medicine Peking University Third Hospital Beijing China; ^3^ Beijing Institute of Heart Lung and Blood Vessel Diseases Beijing Anzhen Hospital Capital Medical University Beijing China; ^4^ Department of Endocrinology Peking University First Hospital Beijing China; ^5^ Shepton High School Plano Texas USA; ^6^ Department of Endocrinology and Metabolism, State Key Laboratory of Female Fertility Promotion Peking University Third Hospital Beijing China; ^7^ Department of General Surgery Peking University Third Hospital Beijing China; ^8^ Department of Human Anatomy and Histology and Embryology, School of Basic Medical Sciences Peking University Beijing China; ^9^ Research Unit of Medical Science Research Management/Basic and Clinical Research of Metabolic Cardiovascular Diseases Chinese Academy of Medical Sciences Beijing China; ^10^ Beijing Key Laboratory of Cardiovascular Receptors Research Peking University Third Hospital Beijing China

**Keywords:** kinesin family member 13B, lipoprotein receptor‐related protein 1, metabolic disorders, obesity, peroxisome proliferator‐activated receptor gamma

## Abstract

Obesity is driven by maladaptive adipose expansion, yet the intracellular machinery linking motor protein‐associated intracellular regulation to adipogenic transcription remains incompletely understood. Here, we identify adipocyte‐derived kinesin family member 13B (KIF13B) as a regulator of obesity‐associated metabolic dysfunction through lipoprotein receptor‐related protein 1 (LRP1)/peroxisome proliferator‐activated receptor γ (PPARγ) signaling. KIF13B expression is markedly upregulated in adipose tissue from humans and mice with obesity. Adipocyte‐specific *Kif13b* deletion protected mice from high‐fat diet‐induced weight gain, adipose expansion and inflammation, dyslipidemia, insulin resistance, and hepatic steatosis, while promoting thermogenic remodeling. In mice with established diet‐induced obesity, adeno‐associated virus serotype 9 (AAV9)‐mediated adipocyte‐targeted KIF13B knockdown improved adipose inflammation, lipid and glucose homeostasis, and liver injury; selected metabolic benefits were also observed in *ob/ob* mice. Mechanistically, KIF13B facilitates PPARγ‐responsive transcriptional activity and adipogenic lipid accumulation via LRP1. This proadipogenic KIF13B/LRP1/PPARγ axis is further supported by human adipose transcriptomic analyses, which reveal positive correlations among its components in obesity and Type 2 diabetes. These findings define a previously unrecognized KIF13B–LRP1–PPARγ axis linking intracellular trafficking to pathological adipocyte remodeling and support adipocyte KIF13B as a potential therapeutic target for obesity‐associated metabolic disease.

## Graphical abstract

Adipocyte‐derived KIF13B promotes LRP1 localization and nuclear LRP1–PPARγ transcriptional activity, thereby enhancing adipogenic gene expression, lipid accumulation, and obesity‐associated metabolic dysfunction. Adipocyte‐specific *Kif13b* deletion disrupts this LRP1/PPARγ axis, suppresses adipogenic programming, reduces adipose expansion, and protects against obesity‐associated metabolic abnormalities.

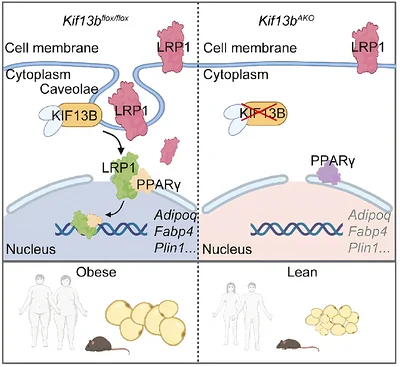

## 1 Introduction

Obesity has emerged as a major global public health challenge, and its prevalence is projected to rise substantially by 2030 [1]. This trend underlies a parallel increase in metabolic diseases, including insulin resistance, Type 2 diabetes (T2DM), metabolic dysfunction‐associated steatotic liver disease (MASLD), cardiovascular disease, and cancer [2, 3, 4, 5, 6]. At its core, obesity is driven by pathological adipose tissue expansion, characterized by adipocyte hypertrophy and hyperplasia. Although current interventions, including lifestyle modification, pharmacotherapies such as glucagon‐like peptide‐1 (GLP‐1) receptor agonists, and bariatric surgery, provide clinical benefit, their long‐term effectiveness is often limited by poor adherence, weight regain, and adverse effects [7, 8, 9, 10]. This therapeutic gap underscores an urgent need to decipher the molecular machinery that governs adipocyte function and adipose tissue remodeling.

Adipocyte differentiation and expansion are energetically demanding processes that necessitate the precise coordination of intracellular membrane trafficking. The delivery of membranes, proteins, and lipids to the plasma membrane and the expansion of lipid droplets relies heavily on the cytoskeletal network and its associated motor proteins [11]. Among these, the kinesin superfamily proteins (KIFs) have recently emerged as critical, yet underexplored, regulators of metabolic homeostasis [12, 13, 14, 15]. Specifically, kinesin family member 13B (KIF13B), a member of the kinesin‐3 family best known for its role in neuronal vesicle transport [16, 17], has garnered attention for its emerging metabolic functions. Independent studies have demonstrated that KIF13B modulates cholesterol metabolism via caveolae‐dependent endocytosis of lipoprotein receptor‐related protein 1 (LRP1) in hepatocytes and protects against metabolic‐associated fatty liver disease (MAFLD) through AMP‐activated protein kinase α1 (AMPKα1) signaling [18, 19]. However, despite the profound reliance of adipocytes on membrane trafficking for lipid droplet biogenesis and hormone responsiveness [20], the specific role of KIF13B in adipose tissue biology and its contribution to obesity pathogenesis remain completely unexplored.

LRP1 is a multifunctional endocytic receptor abundantly expressed in adipocytes, where it governs lipid metabolism and systemic energy balance [21, 22, 23, 24]. Beyond its canonical role as a membrane receptor, emerging evidence indicates that LRP1 can directly interact with the master adipogenic transcription factor peroxisome proliferator‐activated receptor gamma (PPARγ) and translocate to the nucleus to function as a transcriptional coregulator in endothelial cells [25]. Although PPARγ activity is regulated by lipid ligands and post‐translational modifications, whether it integrates signals from cytoskeletal transport machinery remains unknown. Given that LRP1 can act as a PPARγ‐interacting transcriptional coactivator in nonadipose cells and that KIF13B regulates LRP1 trafficking in other metabolic tissues, we herein hypothesized that KIF13B‐dependent regulation of LRP1 may facilitate PPARγ nuclear function in adipocytes, thereby linking cytoskeletal transport to transcriptional control of adipogenesis.

In the present study, we demonstrate that KIF13B is markedly upregulated during adipogenesis and in obesity. Using adipocyte‐specific *Kif13b* deletion mice, we reveal that loss of KIF13B protects against diet‐induced obesity (DIO), insulin resistance, systemic inflammation, and hepatic steatosis. Mechanistically, we uncover that KIF13B promotes LRP1‐mediated activation of PPARγ‐driven adipogenic programs. Therapeutic knockdown of adipocyte‐derived KIF13B by adeno‐associated virus 9 (AAV9)‐*Adipoq‐shKif13b* effectively attenuates established metabolic dysfunction in genetic and diet‐induced obese mice. These findings establish a previously unrecognized axis linking cytoskeletal transport to transcriptional regulation of adipogenesis and identify adipocyte KIF13B as a potential therapeutic target for obesity‐related metabolic disease.

## 2 Results

### 2.1 KIF13B Is Upregulated in Adipose Tissue From Obese Humans and Mice

**FIGURE 1:**
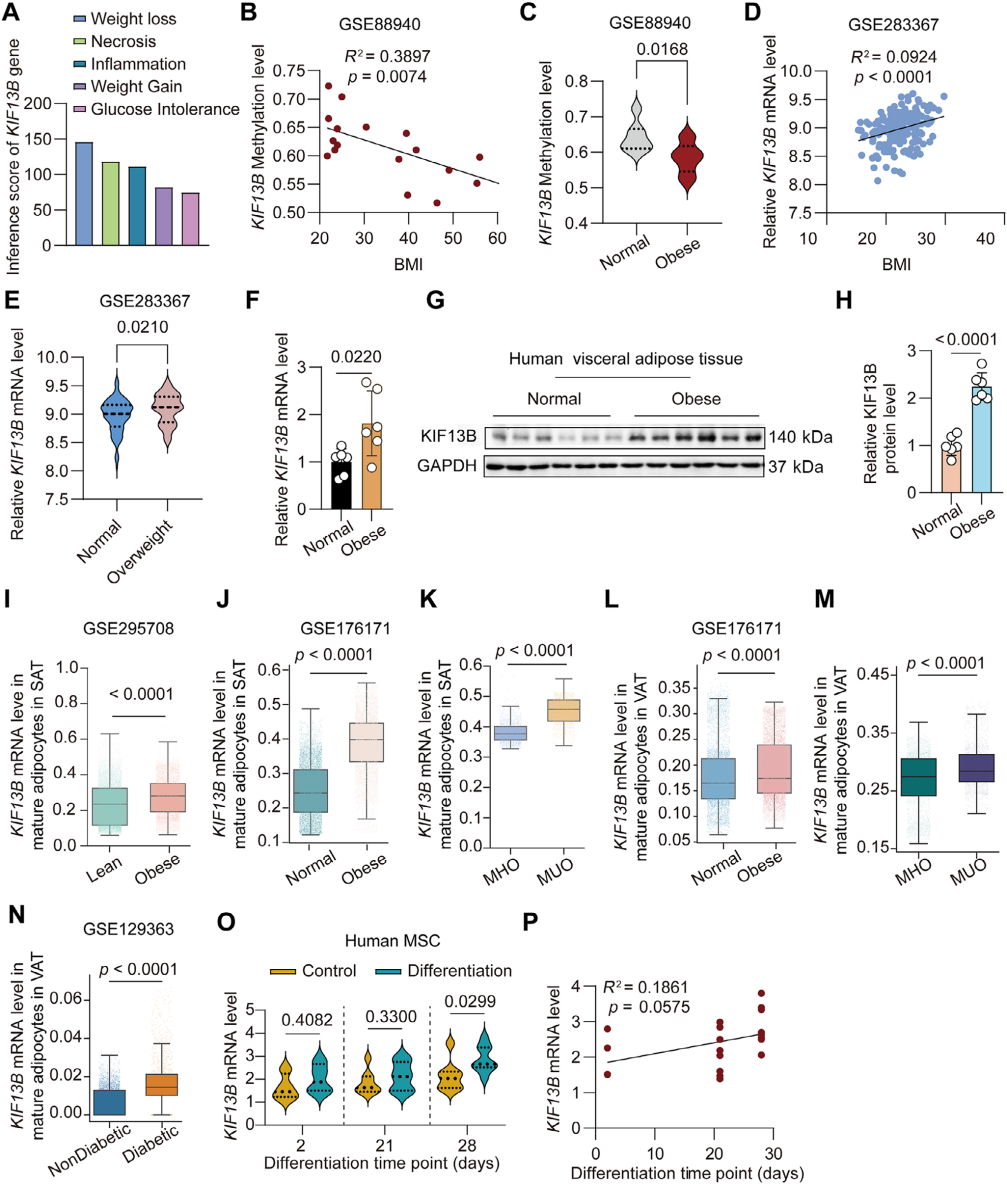
KIF13B expression is increased in human adipose tissue in obesity and during adipogenic differentiation. (A) Comparative Toxicogenomics Database analysis showing kinesin family member 13B‐associated disease/phenotype terms, including body weight. (B) Correlation analysis between *KIF13B* DNA methylation levels in human visceral adipose tissue (VAT) and body mass index (BMI) using the Gene Expression Omnibus (GEO) dataset GSE88940 (*n* = 17). (C) *KIF13B* methylation levels in VAT from normal‐weight and obese individuals from GSE88940 (normal, BMI: 18.5–24.9, *n* = 7; obese, BMI > 30, *n* = 10). (D) Correlation analysis between *KIF13B* mRNA levels in human adipose tissue and BMI using the GSE283367 dataset (*n* = 176). (E) *KIF13B* mRNA levels in adipose tissue from normal‐weight and overweight individuals from GSE283367 (normal, BMI: 18.5–24.9, *n* = 117; overweight, BMI: 25–29.9, *n* = 42). (F) *KIF13B* mRNA levels in VAT from normal and obese subjects in the clinical cohort (*n* = 6 per group). (G, H) Representative western blotting (G) and quantification (H) of KIF13B protein expression in VAT from normal and obese subjects (*n* = 6 per group). (I–K) Single‐nucleus RNA‐sequencing (snRNA‐seq) analysis of *KIF13B* mRNA expression in mature adipocytes from human subcutaneous adipose tissue (SAT), including lean versus obese individuals from GSE295708 (*n* = 6 per group) (I), normal‐weight versus obese individuals from GSE176171 (normal, *n* = 5; obese, *n* = 8) (J), and metabolically healthy obesity (MHO, *n* = 32) versus metabolically unhealthy obesity (MUO, *n* = 45) groups (K). (L–N) SnRNA‐seq analysis of KIF13B expression in mature adipocytes from human VAT, including normal‐weight versus obese individuals (L), MHO versus MUO individuals (M), and nondiabetic versus diabetic individuals from GSE129363 (*n* = 6 per group) (N). GEO dataset GSE135775 contains bulk RNA‐sequencing data from human bone marrow‐derived mesenchymal stromal cells (MSCs) undergoing in vitro adipogenic differentiation. These MSCs were obtained from eight healthy female donors, used at Passage 3, and differentiated according to the previously published Finnish Red Cross Blood Service (FRCBS) protocol. Briefly, cells were first grown to 70%–90% confluence, then exposed to adipogenic induction medium containing 0.1 mM indomethacin (Sigma), 0.5 µg/mL insulin, 0.2 mM 3‐isobutyl‐1‐methylxanthine (IBMX), and 0.4 µg/mL dexamethasone (preadipocyte differentiation medium supplement pack, PromoCell), then the media were changed to the terminal differentiation cocktail containing 0.1 mM indomethacin (Sigma), 0.5 µg/mL insulin, and 3 µg/mL Ciglitazone (PromoCell). Samples were collected at nondifferentiated, early induction (2 days, *n* = 4 independent biological replicates), 3‐week differentiation (*n* = 8 independent biological replicates), and 4‐week differentiation (*n* = 8 independent biological replicates) stages. (O) *KIF13B* mRNA levels during adipogenic differentiation of human mesenchymal stem cells (MSCs) using the GSE135775 dataset. (P) Correlation analysis of *KIF13B* mRNA levels with adipogenic differentiation stage in the dataset shown in (O). All the data are presented as the mean ± standard errors of the mean (SEM). In panels (C), (E), (F), and (H), *p* values were determined via unpaired Student's *t*‐tests. In panels (I), (J), (K), (L), (M), and (N) the two‐tailed Wilcoxon rank‐sum test was performed to calculate the *p* values. In panel (O), multiple unpaired *t*‐tests were carried out to determine the *p* value. In panels (B), (D), and (P), *p* values were computed via simple linear regression.

To investigate the potential association between KIF13B and obesity, we first analyzed the Comparative Toxicogenomics Database, a public resource that integrates curated gene–disease relationships [26], which identified body weight as one of the obesity‐related traits associated with *KIF13B* based on the inference score (Figure 1A). We next examined the epigenetic and transcriptional features of KIF13B in human visceral adipose tissue (VAT). *KIF13B* methylation levels were negatively associated with body mass index (BMI) and were lower in individuals with obesity than in normal controls (Figure 1B,C). In contrast, *KIF13B* mRNA levels in VAT were positively associated with BMI and were higher in overweight individuals than in normal‐weight controls (Figure 1D,E). Consistent with these public dataset analyses, our clinical cohort showed increased *KIF13B* mRNA and protein levels in VAT from patients with obesity compared with normal controls (Figure 1F–H).

To further define the cellular distribution of KIF13B in adipose tissue, we analyzed single‐cell RNA‐sequencing (RNA‐seq) datasets from human adipose depots. *KIF13B* transcripts were detectable across multiple adipose‐resident cell populations, including mature adipocytes, adipose progenitors, immune cells, endothelial cells, and other stromal vascular cells in subcutaneous adipose tissue (SAT; Figure S1A–F) and VAT (Figure S2A–F). In our analyses, the obesity‐associated increase in KIF13B expression was most evident in mature adipocytes. In SAT, mature adipocytes from individuals with obesity or metabolically unhealthy obesity (MUO) showed higher *KIF13B* mRNA expression than those from lean, normal, or metabolically healthy obesity (MHO) individuals (Figure 1I–K). In parallel, mature adipocytes from VAT of individuals with obesity, MUO, or diabetes exhibited higher KIF13B expression than those from normal‐weight, MHO, or nondiabetic counterparts, respectively (Figure 1L–N). In line with these observations, *KIF13B* mRNA levels increased during adipogenic differentiation of human bone marrow‐derived mesenchymal stromal cells (MSCs), suggesting that *KIF13B* expression is associated with adipocyte differentiation (Figure 1O,P).

**FIGURE 2:**
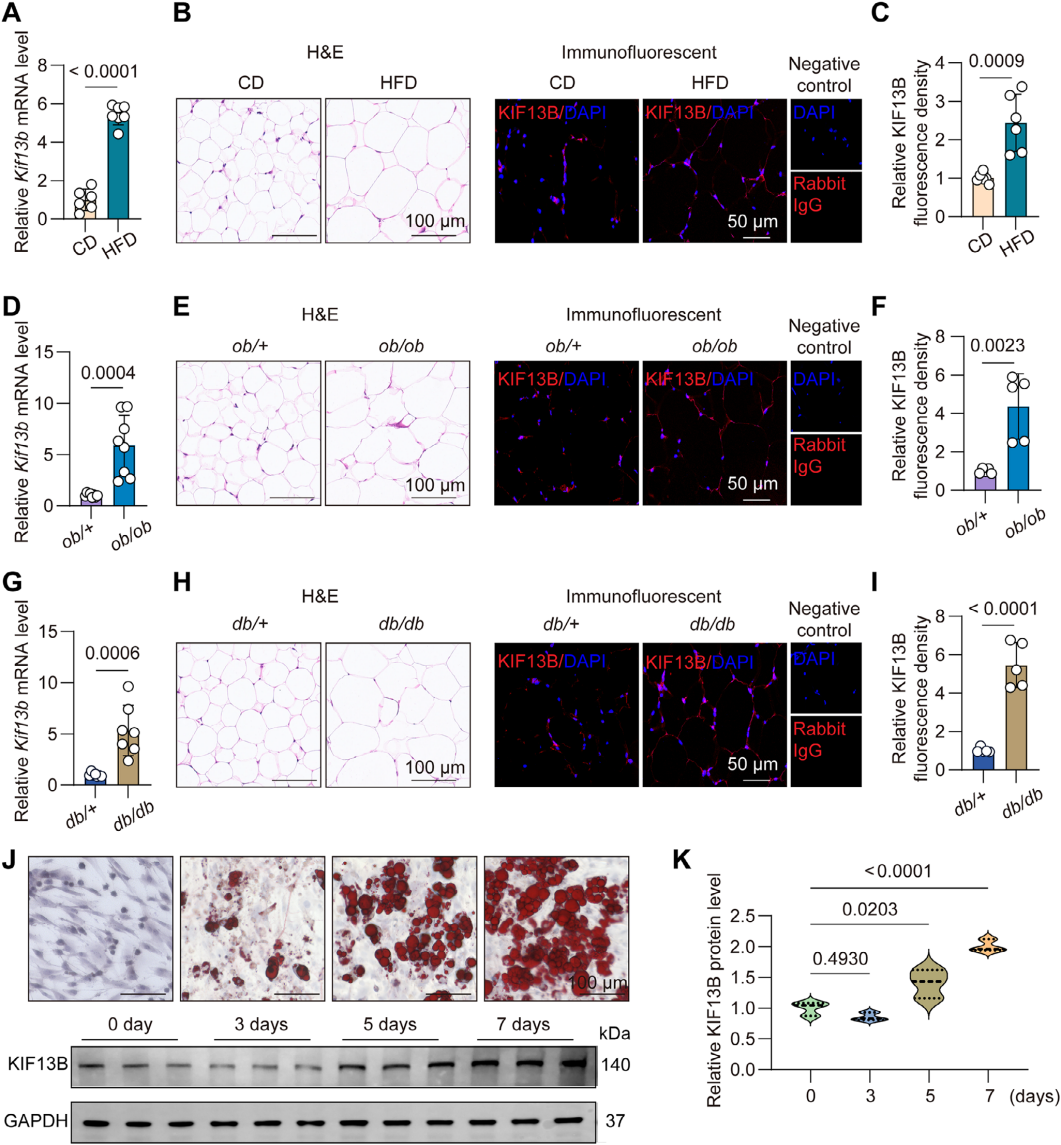
KIF13B expression is increased in adipose tissue from obese mouse models and during adipocyte differentiation in vitro. Eight‐week‐old male wild‐type (WT) mice were fed a high‐fat diet (HFD; research diets, D12492, 60 kcal% fat) for 12 weeks to establish the diet‐induced obesity model. Eight‐week‐old male *ob/ob* and *db/db* mice, and their corresponding control mice on the same genetic background, were maintained on a chow diet for 8 weeks before adipose tissue collection and analysis. (A) *Kif13b* mRNA levels in epididymal white adipose tissue (eWAT) from wild‐type (WT) mice fed an HFD (*n* = 7) or control diet (*n* = 6). (B) Representative hematoxylin and eosin (H&E) staining (left) and immunofluorescence staining of KIF13B (right) in eWAT from WT mice fed an HFD or control diet. (C) Quantification of KIF13B fluorescence intensity in eWAT from HFD‐fed and control WT mice (*n* = 6 per group). (D) *Kif13b* mRNA levels in eWAT from *ob/ob* mice and control mice (*n* = 8 per group). (E) Representative H&E staining (left) and immunofluorescence staining of KIF13B (right) in eWAT from *ob/ob* mice and control mice. (F) Quantification of KIF13B fluorescence intensity in eWAT from *ob/ob* mice and controls (*n* = 5 per group). (G) *Kif13b* mRNA levels in eWAT from *db/db* mice and control mice (*n* = 7 per group). (H) Representative H&E staining (left) and immunofluorescence staining of KIF13B (right) in eWAT from *db/db* mice and control mice. (I) Quantification of KIF13B fluorescence intensity in eWAT from *db/db* mice and controls (*n* = 5 per group). SVF cells were isolated from 6‐week‐old male mouse eWAT by collagenase II digestion, cultured to confluence, and then induced to differentiate into adipocytes using adipogenic medium containing 10 µg/mL insulin, 0.5 mM 3‐isobutyl‐1‐methylxanthine, 1 µM dexamethasone, and 2.5 µM rosiglitazone and incubated for 2 days. Then, the cells were switched to maintenance medium (Dulbecco's modified Eagle medium/Ham's F‐12 [DMEM/F‐12] containing 10% fetal bovine serum [FBS], 1% penicillin/streptomycin [P/S], and 10 µg/mL insulin) for 2 days, followed by replacement with fresh maintenance medium and incubation for another 2 days. Cells were collected at Days 0, 3, 5, and 7 during differentiation for oil red O staining and western blot analysis. (J) Representative oil red O staining of primary mouse stromal vascular fraction (SVF) cells differentiated into adipocytes (up) and western blotting of KIF13B protein (*n* = 3 independent biological replicates). (K) Quantification of KIF13B protein expression during differentiation of mouse SVF cells into adipocytes. Data are presented as mean ± SEM. *p* values in panels (A), (C), (D), (F), (G), and (I) were determined by unpaired Student's *t*‐test. *p* values in panel (K) were determined by one‐way analysis of variance (ANOVA) followed by Tukey's multiple‐comparisons test.

We subsequently evaluated the evolutionary conservation of this obesity‐associated expression pattern in murine models. *KIF13B* mRNA and protein expression were significantly increased in epididymal white adipose tissue (eWAT) from diet‐induced obese mice (Figure 2A–C) and from genetic models of obesity, including *ob/ob* (Figure 2D–F) and *db/db* mice (Figure 2G–I). Furthermore, KIF13B expression progressively increased during the differentiation of primary mouse stromal vascular fraction (SVF) cells into mature adipocytes (Figure 2J,K).

Collectively, these data indicate that KIF13B expression levels are elevated in adipose tissue in obesity across human datasets, clinical samples, and mouse models, and that its expression increases during adipogenic differentiation. These findings support an association between KIF13B and adipose tissue remodeling in obesity.

### 2.2 Adipocyte‐Specific *Kif13b* Deletion Prevents Diet‐Induced Obesity and Promotes Thermogenic Remodeling

To explore the functional contribution of adipocyte KIF13B to obesity and metabolic dysfunction, we generated adipocyte‐specific *Kif13b* knockout mice by crossing *Kif13b^flox/flox^* mice with *Adipoq‐Cre* mice, hereafter referred to as *Kif13b^AKO^* (Figure S3A). Efficient and selective deletion of *Kif13b* in adipose tissues was confirmed by quantitative polymerase chain reaction (qPCR) analysis. Compared with *Kif13b^flox/flox^* littermate controls, *Kif13b^AKO^* mice showed a marked reduction in *Kif13b* mRNA levels in eWAT and brown adipose tissue (BAT), whereas *Kif13b* expression was largely unchanged in nonadipose tissues, including lung, testis, heart, brain, skeletal muscle, liver, and jejunum (Figure S3B). Consistently, KIF13B protein abundance was substantially decreased in both eWAT and BAT from *Kif13b^AKO^* mice, as assessed by western blotting (Figure S3C). Immunofluorescence staining further confirmed the efficient loss of KIF13B signal in eWAT, inguinal white adipose tissue (iWAT), and BAT, with minimal background staining in immunoglobulin G (IgG) negative controls (Figure S3D–G). Together, these data validate the successful generation of an adipocyte‐specific *Kif13b* knockout mouse model.

**FIGURE 3:**
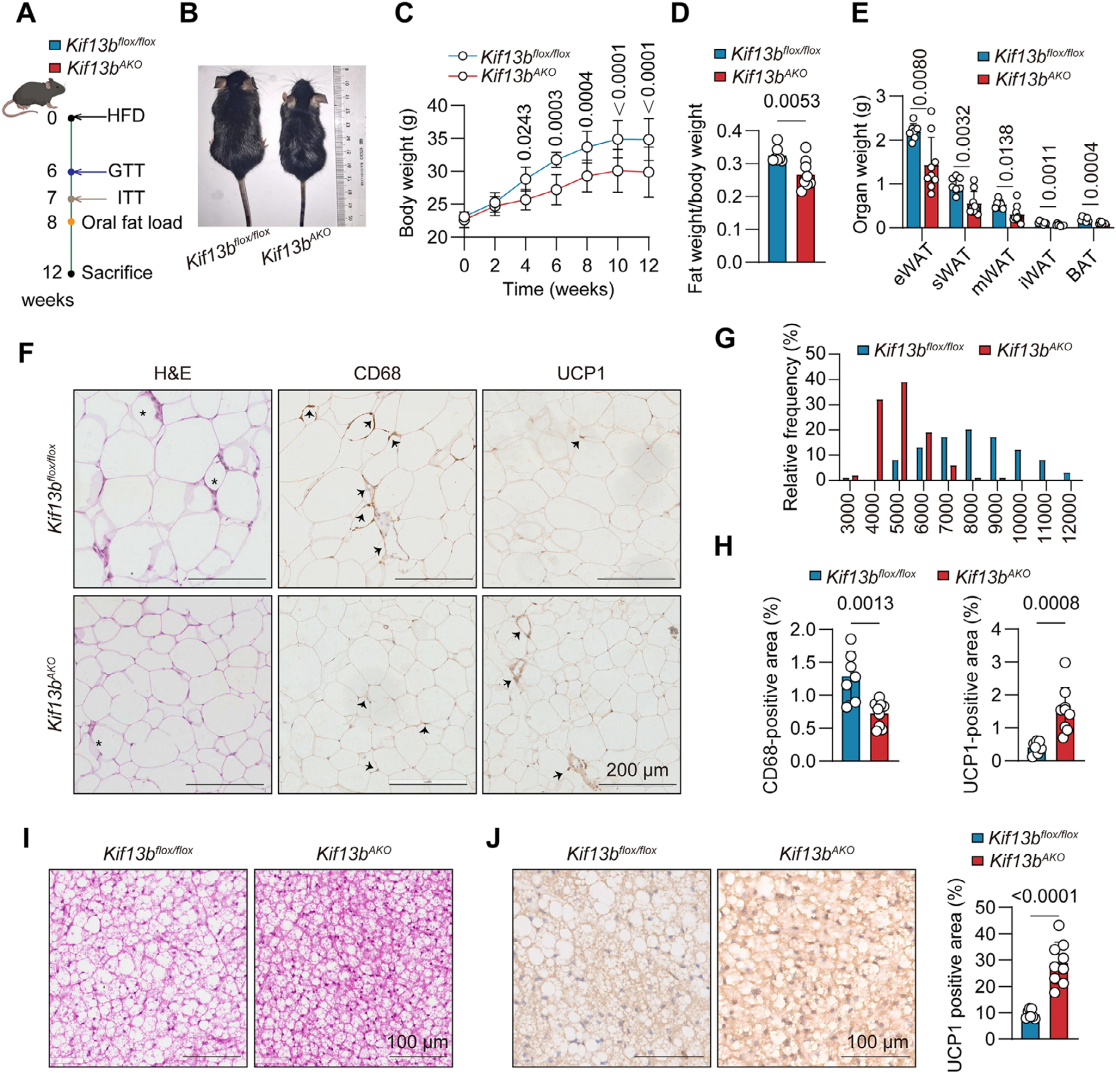
Adipocyte‐specific *Kif13b* deletion attenuates HFD‐induced adiposity and adipose tissue remodeling. (A) Schematic diagram of the HFD‐feeding experiment in *Kif13b^flox/flox^* and *Kif13b^AKO^* mice. Eight‐week‐old male mice were fed an HFD for 12 weeks. (B) Gross appearance of *Kif13b^flox/flox^* and *Kif13b^AKO^* mice at the time of sacrifice. (C) Body weight during HFD feeding (*Kif13b^flox/flox^*, *n* = 10; *Kif13b^AKO^*, *n* = 9). (D) Whole‐body fat mass or fat mass normalized to body weight in HFD‐fed *Kif13b^flox/flox^* (*n* = 7) and *Kif13b^AKO^* (*n* = 9) mice. (E) Weights of major adipose depots, including eWAT, subcutaneous white adipose tissue (sWAT), mesenteric white adipose tissue (mWAT), inguinal white adipose tissue (iWAT), and brown adipose tissue (BAT). (F) Representative H&E staining and immunohistochemical staining for CD68 and UCP1 in eWAT from HFD‐fed mice. Asterisks indicate crown‐like structures; black arrows indicate cluster of differentiation 68 (CD68)‐ or uncoupling protein 1 (UCP1)‐positive staining. (G) Adipocyte size analysis in eWAT (*Kif13b^flox/flox^*, *n* = 5; *Kif13b^AKO^*, *n* = 7). For each mouse, 100 randomly selected fields of view were analyzed. (H) Quantification of CD68‐positive and UCP1‐positive areas in eWAT. (I) Representative H&E staining in BAT. (J) Representative UCP1 immunohistochemical staining and quantification of UCP1‐positive area in BAT. (E, H, I) *Kif13b^flox/flox^*, *n* = 7; *Kif13b^AKO^*, *n* = 9. Data are presented as mean ± SEM. In panels (D), (H), and (J), *p* values were determined by unpaired Student's *t*‐test. For panel (C), *p* value was analyzed by two‐way ANOVA followed by Tukey's multiple‐comparisons test. For panel (E), *p* values were analyzed by multiple unpaired *t*‐tests.

When challenged with a high‐fat diet (HFD) for 12 weeks, *Kif13b^AKO^* mice gained less body weight and exhibited reduced body weight‐normalized fat mass compared with *Kif13b^flox/flox^* littermate controls (Figure 3A–D). Consistently, the weights of major white adipose depots, including eWAT, subcutaneous white adipose tissue (sWAT), mesenteric white adipose tissue (mWAT), iWAT, and BAT, were lower in *Kif13b^AKO^* mice (Figure 3E). Histological analysis of eWAT revealed smaller adipocytes, reduced cluster of differentiation 68 (CD68)‐positive macrophage accumulation, and increased uncoupling protein 1 (UCP1) staining in HFD‐fed *Kif13b^AKO^* mice (Figure 3F–H).

In BAT, *Kif13b^AKO^* mice displayed a more multilocular morphology and markedly higher UCP1 expression than *Kif13b^flox/flox^* mice (Figure 3I,J). These findings indicate that adipocyte‐specific *Kif13b* deletion attenuates HFD‐induced white adipose expansion and promotes thermogenic remodeling of adipose tissues. Consistent with these tissue‐level observations, Seahorse analysis of SVF‐derived adipocytes showed that *Kif13b* deficiency increased mitochondrial oxygen consumption, including basal respiration, ATP‐linked respiration and maximal respiration (Figure S4A,B). These data suggest that adipocyte *Kif13b* deletion enhances adipocyte oxidative phosphorylation and mitochondrial oxidative metabolism.

We next examined whether these adipose tissue and cellular phenotypes were associated with changes in whole‐body energy metabolism and found that HFD‐fed *Kif13b^AKO^* mice showed a modest but significant increase in core body temperature compared with *Kif13b^flox/flox^* controls, whereas daily food intake was not significantly different between the two genotypes (Figure S5A,B). Body weight‐normalized indirect calorimetry showed higher oxygen consumption (VO_2_), carbon dioxide production (VCO_2_), and energy expenditure (EE) in *Kif13b^AKO^* mice, as reflected by increased area under the curve (AUC) values of VO_2_, VCO_2_, and EE (Figure S5C,D,G). In contrast, the respiratory exchange ratio (RER) and physical activity were not significantly altered, although both showed a trend toward increase in *Kif13b^AKO^* mice (Figure S5E,F).

Because *Kif13b^AKO^* mice had markedly lower body weight after HFD feeding, we further analyzed raw, unnormalized metabolic parameters. Raw VO_2_ was lower in *Kif13b^AKO^* mice during the light phase and over the total recording period, while raw VCO_2_ was modestly reduced during the light phase but not significantly changed over the total period (Figure S6A,B). Raw EE was also lower in *Kif13b^AKO^* mice during the light phase and over the total period, whereas raw RER and activity counts did not differ significantly between genotypes (Figure S6C–E). Moreover, analysis of covariance (ANCOVA) of raw VO_2_, VCO_2_, and EE using body weight as a covariate did not identify a significant genotype effect (Figure S6A–E and Table S5). Therefore, although adipocyte‐specific *Kif13b* deletion promotes thermogenic remodeling and enhances adipocyte oxidative metabolism, the indirect calorimetry data do not support a clear body weight‐independent increase in whole‐body oxygen consumption or EE under HFD conditions.

### 2.3 Kif13b Ablation in Adipocytes Improves Systemic Metabolism and Prevents Hepatic Injury

**FIGURE 4:**
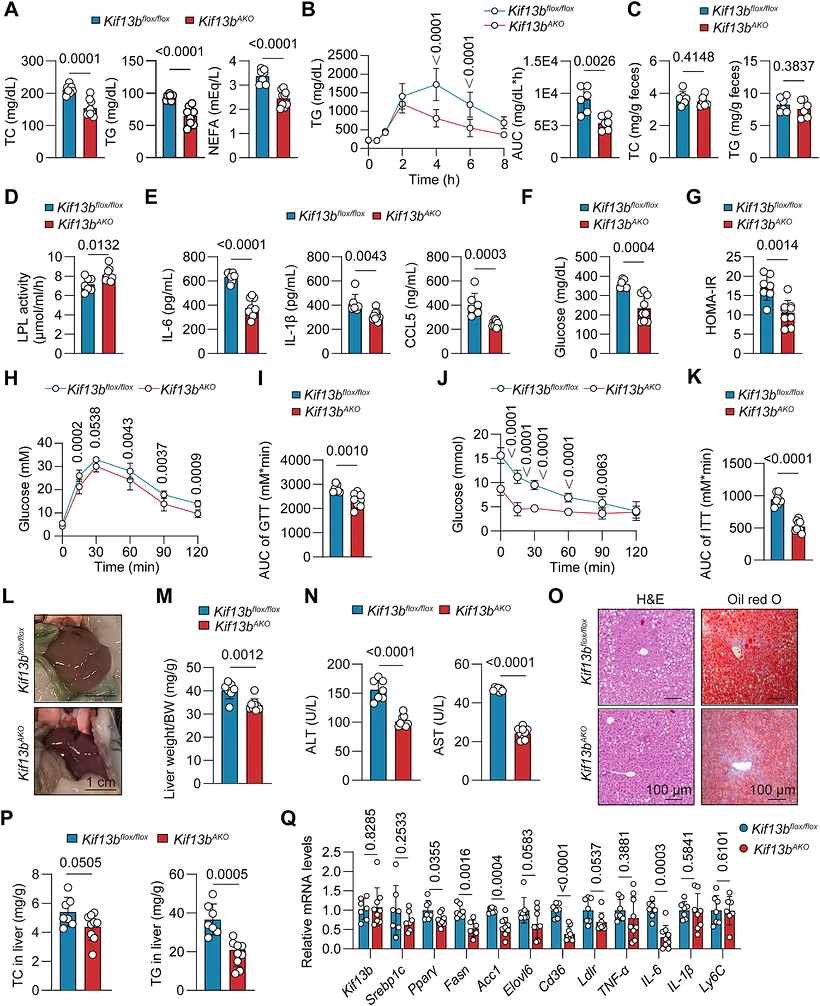
Adipocyte‐specific *Kif13b* deletion improves systemic metabolic parameters and attenuates hepatic lipid accumulation in HFD‐fed mice. (A) Fasting plasma total cholesterol (TC), triglyceride (TG), and nonesterified fatty acid (NEFA) levels in HFD‐fed *Kif13b^flox/flox^* and *Kif13b^AKO^* mice. (B) Oral lipid tolerance test and area under the curve (AUC) analysis after oral lipid challenge (*n* = 6 per group). (C) Fecal TC and TG contents (*n* = 6 per group). (D) Postheparin plasma lipoprotein lipase (LPL) activity. (E) Plasma interleukin‐6 (IL‐6), interleukin‐1β (IL‐1β), and C–C motif chemokine ligand 5 (CCL5) levels. (F) Fasting plasma glucose levels. (G) Homeostatic model assessment of insulin resistance (HOMA‐IR). (H, I) Glucose tolerance test (GTT) (H) and AUC analysis (I). (J, K) Insulin tolerance test (ITT) (J) and AUC analysis (K). (L) Gross appearance of livers from HFD‐fed *Kif13b^flox/flox^* and *Kif13b^AKO^* mice. (M) Liver weight normalized to body weight. (N) Plasma alanine aminotransferase (ALT) and aspartate aminotransferase (AST) levels. (O) Representative H&E and oil red O staining of liver frozen sections. Scale bars, 100 µm. (P) Hepatic TC and TG contents. (Q) Relative mRNA levels of genes related to hepatic de novo lipogenesis, lipid uptake, and inflammation. (A, D–K, M, N, and P, Q) *Kif13b^flox/flox^*, *n* = 7; *Kif13b^AKO^*, *n* = 9. Data are presented as mean ± SEM. In panels (A), (C), (D), (E), (F), (G), (I), (K), (M), (N), and (P), *p* values were determined via unpaired Student's *t*‐tests. For panel (Q), multiple unpaired *t*‐tests were carried out to determine the *p* value. For panels (B), (H), and (J), *p* values were analyzed by two‐way ANOVA followed by Tukey's multiple‐comparisons test. For panel (B), AUC was analyzed by unpaired Student's *t*‐test.

We next assessed systemic metabolic parameters in HFD‐fed mice. *Kif13b^AKO^* mice displayed lower plasma levels of total cholesterol (TC), triglycerides (TGs), and nonesterified fatty acid (NEFA; Figure 4A). In an oral lipid tolerance test, plasma TG levels were lower in *Kif13b^AKO^* mice during the postprandial phase, with a reduced AUC indicating improved postprandial TG handling (Figure 4B). Fecal TC and TG content were not significantly different between the two genotypes (Figure 4C), suggesting that adipocyte‐specific *Kif13b* deletion does not measurably increase fecal lipid loss under these conditions. In contrast, postheparin plasma lipoprotein lipase (LPL) activity was higher in *Kif13b^AKO^* mice (Figure 4D), suggesting that increased peripheral TG handling may contribute to the improved lipid profile. Plasma concentrations of interleukin‐6 (IL‐6), interleukin‐1β (IL‐1β), and C–C motif chemokine ligand 5 (CCL5) were also reduced in *Kif13b^AKO^* mice, consistent with lower systemic inflammatory tone (Figure 4E).

In addition to the improved lipid profile, *Kif13b^AKO^* mice showed lower plasma glucose levels and a reduced homeostatic model assessment of insulin resistance (HOMA‐IR) index compared with *Kif13b^flox/flox^* controls (Figure 4F,G). Glucose tolerance tests (GTTs) further showed lower glucose excursions and a reduced AUC in *Kif13b^AKO^* mice (Figure 4H,I). Similarly, insulin tolerance tests (ITTs) revealed greater insulin responsiveness in *Kif13b^AKO^* mice, as reflected by lower glucose levels after insulin administration and a reduced AUC (Figure 4J,K). Together, these data indicate that adipocyte‐specific *Kif13b* deletion attenuates HFD‐induced adiposity and is associated with improved lipid metabolism, reduced inflammatory markers and better glucose homeostasis.

Given the improvement in adiposity and systemic metabolism, we further assessed hepatic lipid accumulation in HFD‐fed mice. Gross examination showed that livers from *Kif13b^AKO^* mice appeared less pale than those from *Kif13b^flox/flox^* controls (Figure 4L). *Kif13b^AKO^* mice also displayed a lower liver weight‐to‐body weight ratio and reduced plasma alanine aminotransferase (ALT) and aspartate aminotransferase (AST) levels (Figure 4M,N), suggesting attenuated liver enlargement and hepatocellular injury. Histological analysis by hematoxylin and eosin (H&E) and oil red O staining showed reduced hepatic lipid accumulation in *Kif13b^AKO^* mice (Figure 4O). Consistently, hepatic TG content was significantly decreased, whereas hepatic TC content showed a trend toward reduction that did not reach statistical significance (Figure 4P). At the transcriptional level, hepatic expression of genes related to lipogenesis and lipid uptake, including *Pparγ*, *Fasn*, *Acc1*, and *Cd36*, was reduced in *Kif13b^AKO^* mice, whereas *Kif13b*, *Srebp1c*, *Tnf‐α*, *Il‐1β*, and *Ly6c* were not significantly changed. *Elovl6* and *Ldlr* showed a trend toward reduction, and Il‐6 expression was significantly lower in *Kif13b^AKO^* mice (Figure 4Q). Together, these findings indicate that adipocyte‐specific *Kif13b* deletion is associated with reduced hepatic steatosis and liver injury in HFD‐fed mice, accompanied by lower expression of selected hepatic lipogenic, lipid uptake and inflammatory markers.

### 2.4 Therapeutic Knockdown of Adipocyte‐Derived KIF13B Ameliorates Established Metabolic Dysfunction in Obese Mice

**FIGURE 5:**
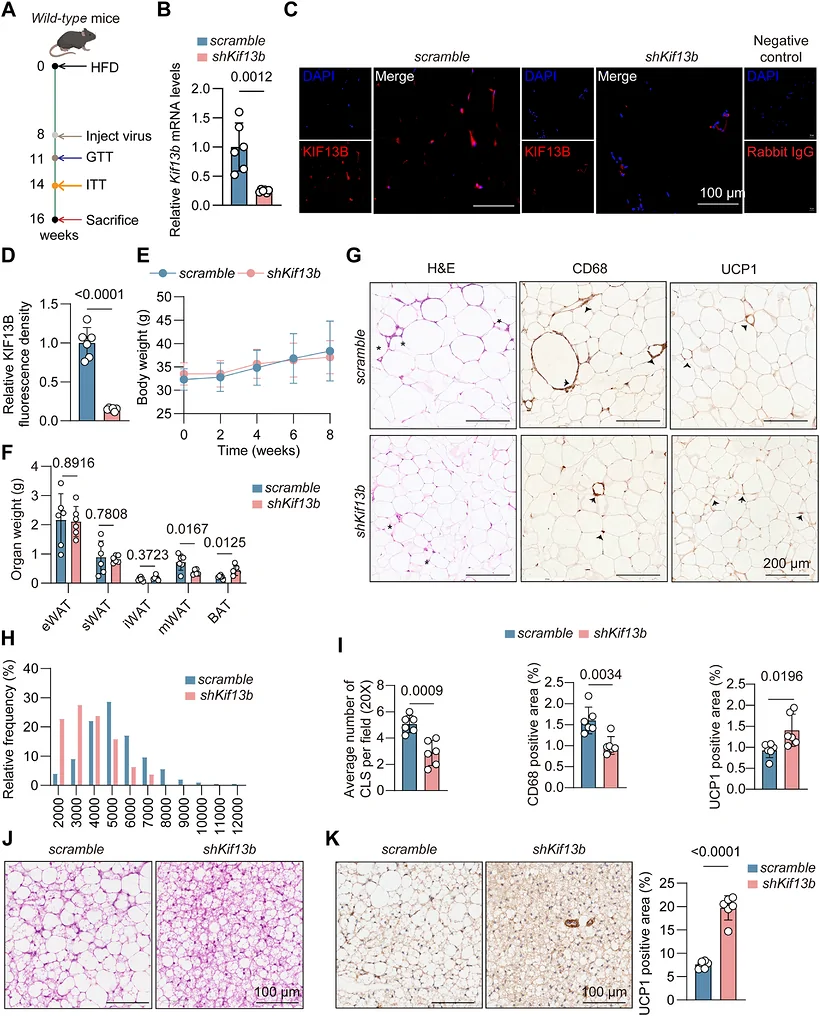
AAV9‐mediated KIF13B knockdown remodels adipose tissue in diet‐induced obese mice. (A) Schematic diagram of adeno‐associated virus serotype 9 (AAV9)‐short hairpin RNA (shRNA) intervention in diet‐induced obese (DIO) mice. Eight‐week‐old male WT mice were fed an HFD for 8 weeks, injected via the tail vein with AAV9‐*Adipoq‐scramble* or AAV9‐*Adipoq‐shKif13b*, and maintained on HFD for an additional 8 weeks. GTT and ITT were performed during the intervention period, and mice were sacrificed at Week 16. (B) Quantitative polymerase chain reaction (qPCR) analysis of *Kif13b* mRNA levels in eWAT after AAV9*‐Adipoq‐shKif13b* treatment. (C, D) Representative KIF13B immunofluorescence staining (C) and quantification of KIF13B fluorescence intensity (D) in adipose tissue. Rabbit immunoglobulin G (IgG) was used as a negative control. (E) Body weight during the treatment period. (F) Weights of eWAT, sWAT, iWAT, mWAT, and BAT. (G) Representative H&E staining and immunohistochemical staining for CD68 and UCP1 in eWAT. Asterisks indicate crown‐like structures; black arrows indicate CD68‐ or UCP1‐positive staining. Scale bars, 200 µm. (H) Adipocyte size distribution in eWAT. For each mouse, 200 randomly selected fields of view were analyzed. (I) Quantification of crown‐like structures (CLSs), CD68‐positive area and UCP1‐positive area in eWAT. (J) Representative H&E staining of BAT. Scale bar, 100 µm. (K) Representative UCP1 immunohistochemical staining and quantification of UCP1‐positive area in BAT. Scale bar, 100 µm. (B, D–F, H, I, K) *n* = 6 per group. Data are presented as mean ± SEM. In panels (B), (D), (I), and (K), *p* values were determined via unpaired Student's *t*‐tests. For panel (E), *p* values were analyzed by two‐way ANOVA followed by Tukey's multiple‐comparisons test. For panel (F), multiple unpaired *t*‐tests were carried out to determine the *p* value.

To further evaluate whether reducing adipose KIF13B expression could improve obesity‐associated metabolic dysfunction, we used an adeno‐associated virus serotype 9 (AAV9)‐*Adipoq*‐short hairpin RNA (shRNA) approach in established DIO mice. Wild‐type (WT) mice were fed an HFD for 8 weeks and subsequently administered AAV9 vectors encoding either *Adipoq*‐*shKif13b* or an *Adipoq*‐*scramble* control via tail intravenous injection. Metabolic phenotyping and tissue collection were performed after an additional 8‐week intervention period (Figure 5A). AAV9‐*Adipoq*‐*shKif13b* markedly reduced *Kif13b* mRNA expression and KIF13B immunofluorescence signal in adipose tissue compared with *scramble* control (Figure 5B–D).

During the treatment period, AAV9‐*Adipoq*‐*shKif13b* did not significantly alter overall body weight in DIO mice (Figure 5E). Among the adipose depots examined, mWAT weight was reduced, whereas BAT weight was increased; eWAT, sWAT, and iWAT weights were not significantly changed (Figure 5F). Histological analysis of eWAT showed a shift toward smaller adipocytes, fewer crown‐like structures (CLSs) and reduced CD68‐positive macrophage accumulation after KIF13B knockdown (Figure 5G–I). In parallel, the UCP1‐positive area was increased in eWAT (Figure 5G,I). BAT from AAV9‐*Adipoq*‐*shKif13b*‐treated mice also showed increased UCP1 staining compared with *scramble* controls (Figure 5J,K). These data suggest that KIF13B knockdown remodels adipose tissue morphology with reduced adipose inflammation and increased expression of thermogenic markers in DIO mice.

**FIGURE 6:**
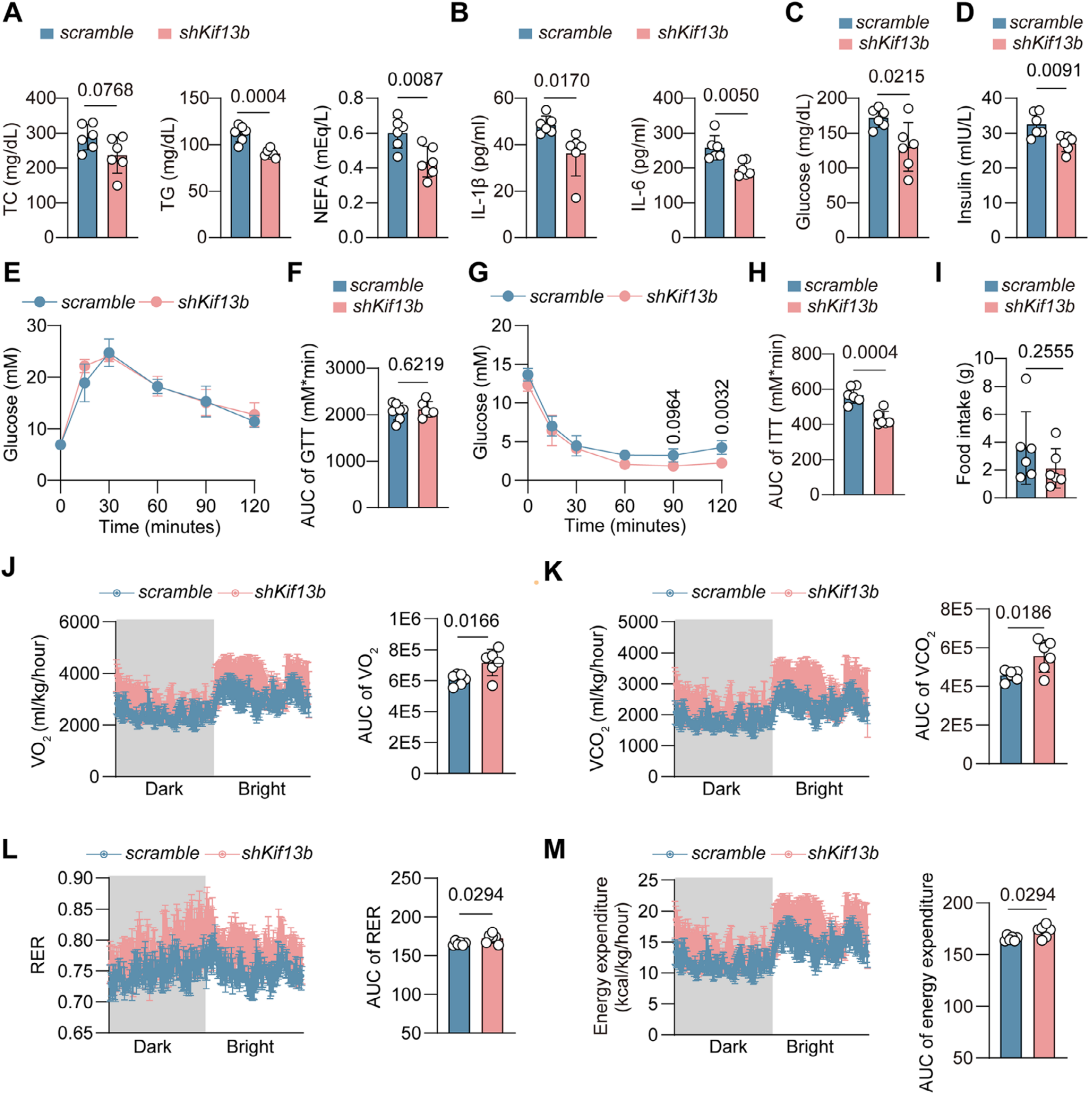
AAV9‐mediated KIF13B knockdown improves selected systemic metabolic parameters in DIO mice. Eight‐week‐old male WT mice were fed an HFD for 8 weeks, injected via the tail vein with AAV9‐*Adipoq*‐*scramble* or AAV9‐*Adipoq*‐*shKif13b*, and maintained on HFD for an additional 8 weeks. (A) Fasting plasma TC, TG, and NEFA levels in DIO mice treated with AAV9*‐Adipoq*‐*scramble* or AAV9‐*Adipoq*‐*shKif13b*. (B) Plasma IL‐1β and IL‐6 levels. (C) Fasting plasma glucose levels. (D) Fasting plasma insulin levels. (E, F) GTT (E) and AUC analysis (F) (AAV9‐*Adipoq*‐*scramble*, *n* = 7; AAV9‐*Adipoq*‐*shKif13b*, *n* = 6). (G, H) ITT (G) and AUC analysis (H) (AAV9‐*Adipoq*‐*scramble*, *n* = 7; AAV9‐*Adipoq*‐*shKif13b*, *n* = 6). (I) Daily food intake. (J–M) Twenty‐four‐hour indirect calorimetry analysis of body weight‐normalized oxygen consumption (VO_2_) (J), carbon dioxide production (VCO_2_) (K), respiratory exchange ratio (RER) (L) and energy expenditure (EE) (M), with corresponding AUC values where indicated. VO_2_, VCO_2_, and EE are presented as body weight‐normalized values exported from the Oxymax software. (A–D and G–M) *n* = 6 per group. Data are presented as mean ± SEM. *p* values for two‐group comparisons and AUCs were determined by unpaired Student's *t*‐test. GTT and ITT time‐course data were analyzed by two‐way ANOVA followed by Tukey's multiple‐comparisons test.

We next examined systemic metabolic parameters and inflammatory factors. AAV9‐*Adipoq*‐*shKif13b* treatment significantly reduced circulating TG and NEFA levels, whereas plasma TC showed a trend toward reduction (Figure 6A). Circulating IL‐1β and IL‐6 levels were also decreased (Figure 6B). In addition, AAV9‐*Adipoq*‐*shKif13b*‐treated mice showed lower fasting plasma glucose and insulin levels (Figure 6C,D). Glucose tolerance was not significantly altered, as shown by comparable glucose excursion curves and unchanged GTT AUC (Figure 6E,F). By contrast, insulin tolerance was improved, with lower glucose levels after insulin administration and a reduced ITT AUC in the AAV9*‐Adipoq‐shKif13b* group (Figure 6G,H). Daily food intake was not significantly different between the two groups (Figure 6I).

Indirect calorimetry showed higher body weight‐normalized VO_2_, VCO_2_, RER, and EE in AAV9‐*Adipoq*‐*shKif13b*‐treated DIO mice (Figure 6J–M). However, when VO_2_, VCO_2_, and EE were analyzed without body weight normalization, these parameters were not significantly different between groups, although RER remained modestly increased (Figure S7A–D). ANCOVA analysis did not detect a significant AAV9‐*Adipoq*‐*shKif13b*‐treated effect on VO_2_, VCO_2_, or EE after body weight adjustment, indicating that KIF13B knockdown did not significantly alter these raw indirect calorimetry parameters independently of body weight (Table S6).

Given the improvement in lipid and glucose metabolic parameters, we next evaluated hepatic lipid accumulation in DIO mice. AAV9‐*Adipoq‐shKif13b* treatment did not significantly change liver weight or the liver weight‐to‐body weight ratio (Figure S8A). Plasma ALT levels were markedly reduced, whereas AST levels were not significantly changed (Figure S8B). H&E and oil red O staining showed reduced hepatic lipid accumulation in AAV9‐*Adipoq‐shKif13b*‐treated mice (Figure S8C). Consistently, hepatic TG content was significantly decreased, while hepatic TC content was not significantly altered (Figure S8D). At the transcriptional level, KIF13B knockdown was associated with reduced hepatic expression of selected de novo lipid synthesis and cholesterol uptake‐related genes, including *Srebp1c*, *Fasn*, *Elovl6*, and *Ldlr*, whereas *Pparγ*, *Acc1*, *Cd36*, and inflammatory markers showed no consistent reduction (Figure S8E). These results indicate that adipose KIF13B knockdown attenuates hepatic TG accumulation and liver injury markers in DIO mice.

We also examined the effect of AAV9‐*Adipoq*‐*shKif13* in *ob/ob* mice, a genetic model of severe obesity (Figure S9A). AAV9‐*Adipoq*‐*shKif13* efficiently reduced *Kif13b* mRNA expression and KIF13B immunofluorescence signal in adipose tissue (Figure S9B–D). KIF13B knockdown did not significantly affect body weight or the weights of major adipose depots in *ob/ob* mice (Figure S9E,F). In eWAT, adipocyte size distribution shifted toward smaller adipocytes, accompanied by a reduced CD68‐positive area and an increased UCP1‐positive area, whereas the reduction in CLS did not reach statistical significance (Figure S9G–I). Increased UCP1 staining was also observed in BAT after KIF13B knockdown (Figure S9J,K).

Systemically, AAV9‐*Adipoq*‐*shKif13* did not significantly change plasma TC, TG, NEFA, or fasting glucose levels in *ob/ob* mice (Figure S10A,C). However, circulating IL‐6, IL‐1β, and CCL5 levels were reduced, and fasting insulin levels were lower in the AAV9‐*Adipoq*‐*shKif13* group (Figure S10B,D). Glucose tolerance remained unchanged, whereas insulin tolerance was improved, as reflected by lower glucose levels during the ITT and a reduced ITT AUC (Figure S10E–H). Body weight‐normalized VO_2_, VCO_2_, RER, and EE were not significantly altered in *ob/ob* mice (Figure S10I–L). Consistently, non‐normalized VO_2_, VCO_2_, RER, and EE showed no significant differences between groups during the dark phase, light phase, or total recording period (Figure S11A–D).

We further examined hepatic parameters in *ob/ob* mice. AAV9‐*Adipoq*‐*shKif13b* treatment did not significantly alter liver weight, and the liver weight‐to‐body weight ratio showed only a nonsignificant trend toward reduction (Figure S12A). Plasma ALT and AST levels were reduced after KIF13B knockdown (Figure S12B). H&E and oil red O staining showed less hepatic lipid accumulation in AAV9‐*Adipoq*‐*shKif13b*‐treated mice (Figure S12C). Hepatic TG content was significantly decreased, whereas hepatic TC content was unchanged (Figure S12D). qPCR analysis showed reduced hepatic expression of *Fasn*, *Acc1*, and *Elovl6*, while *Srebp1c*, *Pparγ*, *Cd36*, *Ldlr*, *Tnf‐α*, *Il‐6*, *Il‐1β*, and *Ly6c* were not significantly changed (Figure S12E).

Together, these data indicate that AAV9‐mediated KIF13B knockdown in adipocytes improves selected metabolic and inflammatory parameters in obese mice. The effects were more pronounced in the DIO model, where KIF13B knockdown generated favorable phenotypes with improved lipid handling, reduced adipose inflammation, increased UCP1 expression and attenuated hepatic steatosis, whereas in *ob/ob* mice the main detectable effects were reduced inflammatory markers, improved insulin tolerance.

### 2.5 KIF13B Modulates PPARγ Transcriptional Programs in Adipocytes

**FIGURE 7:**
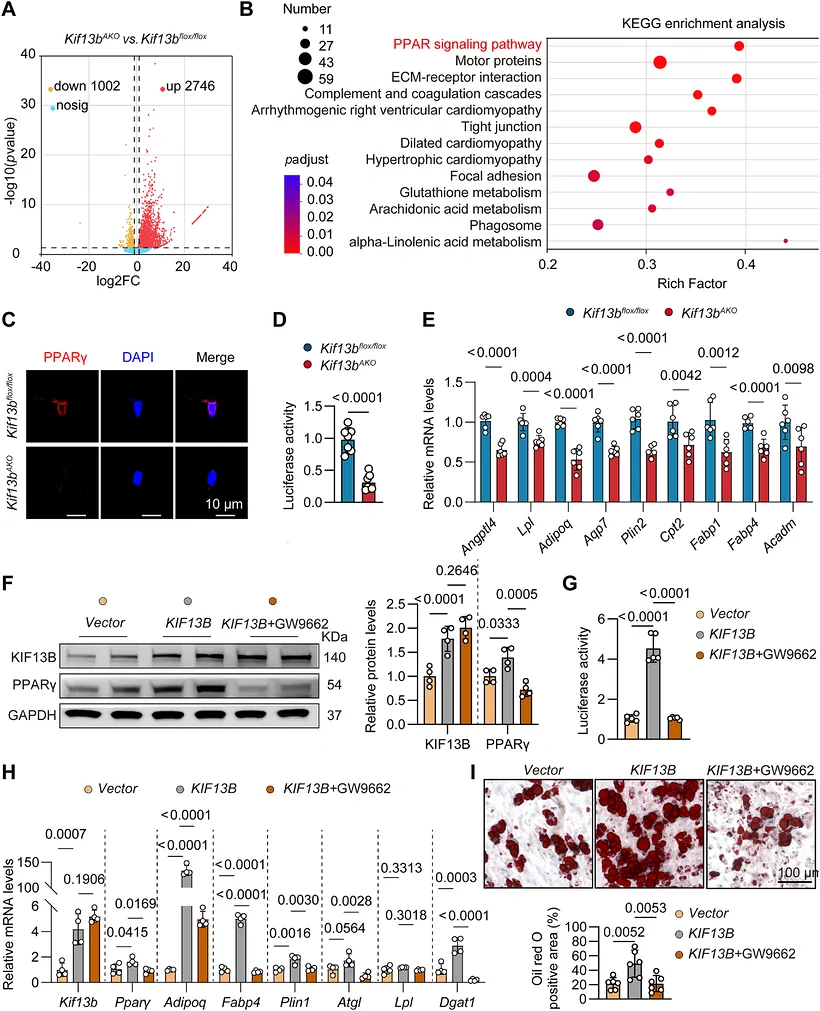
KIF13B modulates PPARγ transcriptional programs in adipocytes. (A) Volcano plot of differentially expressed genes (DEGs) identified by RNA sequencing (RNA‐seq) analysis of adipose tissue from HFD‐fed *Kif13b^flox/flox^* and *Kif13b^AKO^* mice (*n* = 4 per group). (B) KEGG pathway enrichment analysis of DEGs shown in (A). (C) Representative immunofluorescence staining of peroxisome proliferator‐activated receptor gamma (PPARγ) in adipocytes from *Kif13b^flox/flox^* (*n* = 7) and *Kif13b^AKO^* (*n* = 9) mice. Scale bars, 10 µm. (D) Dual‐luciferase reporter PPARγ transcriptional activity in SVF‐derived adipocytes from *Kif13b^flox/flox^* and *Kif13b^AKO^* mice (*n* = 7 independent biological replicates). (E) Relative mRNA levels of PPARγ‐associated adipogenic and lipid metabolism‐related genes in adipose tissue from HFD‐fed *Kif13b^flox/flox^* and *Kif13b^AKO^* mice (*n* = 6 per group). (F) Representative western blotting and quantification of KIF13B and PPARγ protein levels in SVF‐derived adipocytes transfected with the vector, the *KIF13B* plasmid or the *KIF13*B plasmid plus GW9662 (10 nM; *n* = 4 independent biological replicates). (G) Dual‐luciferase reporter PPARγ transcriptional activity in SVFs transfected with the vector, the *KIF13B* plasmid or the *KIF13B* plasmid plus GW9662 (10 nM; *n* = 5 independent biological replicates). (H) Relative mRNA levels of adipogenic and lipid metabolism‐related genes in SVFs transfected with the vector, the *KIF13B* plasmid or the *KIF13B* plasmid plus GW9662 (10 nM; *n* = 4 independent biological replicates). (I) Representative oil red O staining and quantification of lipid accumulation in SVF‐derived adipocytes transfected with the vector, the *KIF13B* plasmid or the *KIF13B* plasmid plus GW9662 (10 nM; *n* = 6 independent biological replicates). Scale bar, 100 µm. Data are presented as mean ± SEM. *p* values in panel (D) were determined by unpaired Student's *t*‐test. *p* values in panel (E) were determined by multiple unpaired *t*‐tests. *p* values in panels (F–I) were determined by one‐way ANOVA followed by Tukey's multiple‐comparisons test.

To investigate the molecular mechanisms through which adipocyte‐specific KIF13B contributes to obesity pathogenesis, we performed RNA‐seq of eWAT from *Kif13b^AKO^* mice and *Kif13b^flox/flox^* controls. This analysis identified 3748 differentially expressed genes (DEGs), including 2746 upregulated and 1002 downregulated genes in *Kif13b^AKO^* mice adipose tissue (Figure 7A). Kyoto Encyclopedia of Genes and Genomes (KEGG) pathway enrichment analysis of these DEGs highlighted several pathways related to adipose tissue remodeling and metabolism, including the PPAR signaling pathway, motor proteins, extracellular matrix (ECM)–receptor interaction, and focal adhesion pathways (Figure 7B). Given the central role of PPARγ in adipocyte differentiation and lipid metabolic gene regulation [27, 28, 29], we next focused on the relationship between KIF13B and PPARγ signaling. Immunofluorescence staining showed reduced PPARγ signal in *Kif13b^AKO^* adipocytes compared with *Kif13b^flox/flox^* controls (Figure 7C). Consistently, PPARγ‐responsive luciferase reporter activity was markedly lower in *Kif13b*‐deficient SVF‐derived adipocytes (Figure 7D). qPCR analysis further showed reduced expression of multiple PPARγ‐associated genes involved in lipid handling and adipocyte function, including *Angptl4*, *Lpl*, *Adipoq*, *Aqp7*, *Plin2*, *Cpt2*, *Fabp1*, *Fabp4*, and *Acadm*, in *Kif13b^AKO^* adipose tissue (Figure 7E). These data indicate that adipocyte *Kif13b* deletion is associated with attenuated PPARγ‐related transcriptional activity.

We then examined whether increasing KIF13B expression was sufficient to enhance PPARγ signaling in adipocytes. KIF13B overexpression increased PPARγ protein abundance and PPARγ‐responsive luciferase activity, whereas treatment with the PPARγ antagonist GW9662 largely blunted the increase in reporter activity without reducing KIF13B overexpression (Figure 7F,G). At the transcriptional level, KIF13B overexpression increased the expression of adipogenic and lipid metabolic genes, including *Pparγ*, *Adipoq*, *Fabp4*, *Plin1*, and *Dgat1*; these changes were partially or largely attenuated by GW9662 (Figure 7H). Consistent with these molecular findings, oil red O staining showed increased lipid accumulation in KIF13B‐overexpressing adipocytes, which was reduced by GW9662 treatment (Figure 7I).

Together, these results suggest that KIF13B is linked to PPARγ‐associated transcriptional activity in adipocytes. Loss of *Kif13b* is accompanied by reduced expression of PPARγ target genes, whereas KIF13B overexpression promotes adipogenic gene expression and lipid accumulation in a manner that is sensitive to pharmacological inhibition of PPARγ.

### 2.6 KIF13B Regulates PPARγ‐Associated Adipogenic Programs Through LRP1

**FIGURE 8:**
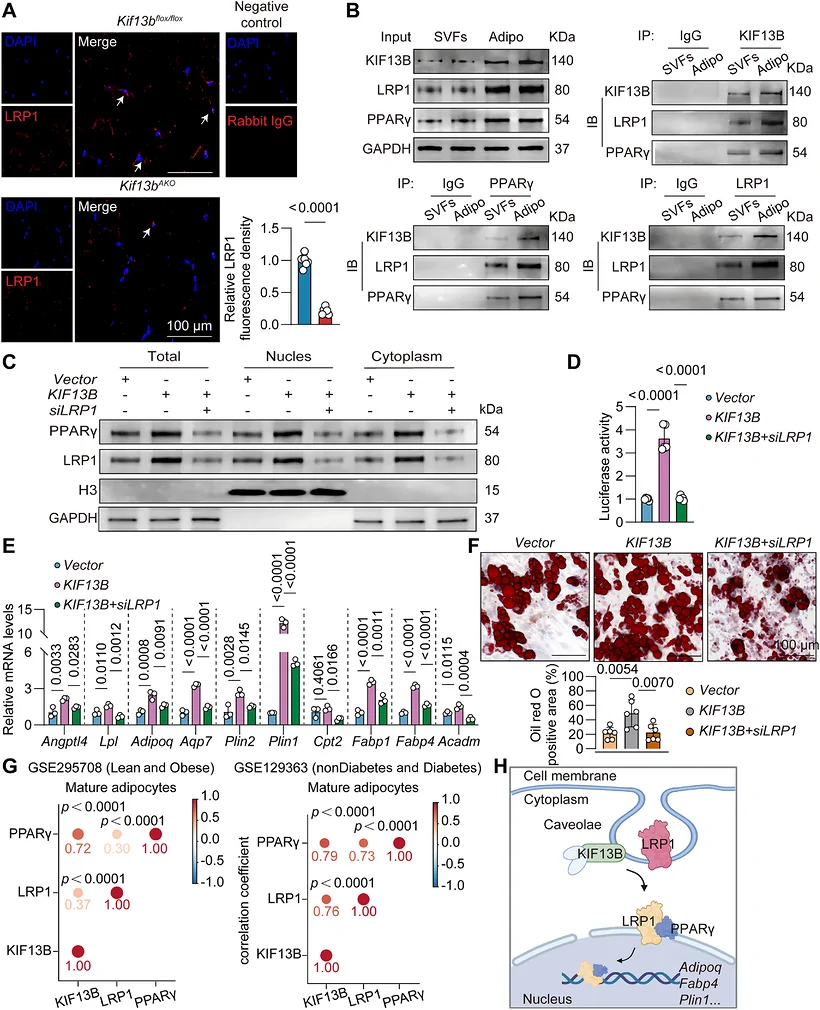
KIF13B regulates PPARγ‐mediated adipogenic programs through LRP1. (A) Representative immunofluorescence staining of low‐density lipoprotein receptor‐related protein 1 (LRP1) in adipocytes from HFD‐fed *Kif13b^flox/flox^* and *Kif13b^AKO^* mice, with quantification of relative LRP1 fluorescence intensity. Rabbit IgG was used as a negative control (*n* = 6 per group). Scale bar, 100 µm. (B) Input and coimmunoprecipitation analyses of KIF13B, LRP1, and PPARγ in stromal vascular fraction (SVF) cells and differentiated adipocytes (adipo). IgG was used as a negative control for immunoprecipitation (*n* = 3 independent biological replicates). (C) Representative western blotting was performed for total, nuclear, and cytoplasmic PPARγ and LRP1 in SVF‐derived adipocytes (*n* = 3 independent biological replicates). These cells were transfected with the vector, the *KIF13B* plasmid, or the *KIF13B* plasmid plus small interfering RNA targeting LRP1 (*siLRP1*). Per lane, 8 µg of whole cell lysate, 15 µg of nuclear, and 10 µg of cytoplasmic total protein were loaded. Histone H3 and GAPDH were used as nuclear and cytoplasmic loading controls, respectively. (D) Dual‐luciferase reporter PPARγ transcriptional activity in SVFs with the vector, the *KIF13B* plasmid, or the *KIF13B* plasmid plus *siLRP1* (*n* = 5 independent biological replicates). (E) Relative mRNA levels of adipogenic and lipid metabolism‐related genes in SVFs transfected with the vector, the *KIF13B* plasmid, or the *KIF13B* plasmid plus *siLRP1* (*n* = 3 independent biological replicates). (F) Representative oil red O staining and quantification of lipid accumulation in SVF‐derived adipocytes transfected with the vector, the *KIF13B* plasmid, or the *KIF13B* plasmid plus *siLRP1* (*n* = 6 independent biological replicates). Scale bar, 100 µm. (G) Spearman correlation analysis of pairwise relationships among KIF13B, LRP1, and PPARγ mRNA levels in human mature adipocytes from GSE295708 (*n* = 6 per group) and GSE129363 (nondiabetes, *n* = 9; diabetes, *n* = 5). (H) Proposed working model in which KIF13B supports LRP1‐associated regulation of PPARγ transcriptional programs during adipocyte differentiation. Data are presented as mean ± SEM. *p* values in panels (D), (E), and (F) were determined by one‐way ANOVA followed by Tukey's multiple‐comparisons test. *p* value in panel (A) was determined by unpaired Student's *t*‐test. *p* values in panel (G) were computed by Spearman correlation analysis.

Given that KIF13B is a microtubule‐associated motor protein primarily involved in intracellular vesicle trafficking, whereas PPARγ functions as a nuclear receptor transcription factor that governs adipocyte metabolic and thermogenic gene programs, we reasoned that KIF13B may regulate PPARγ signaling through an intermediary trafficking‐dependent mechanism rather than through a direct nuclear action. In this context, LRP1 is of particular interest because previous studies have implicated KIF13B in LRP1 trafficking and LRP1 modulated PPARγ activity [18, 25, 30]. We therefore examined whether LRP1 is involved in KIF13B‐associated regulation of PPARγ signaling in adipocytes. Immunofluorescence staining showed that the LRP1 signal was markedly reduced in *Kif13b^AKO^* mice compared with *Kif13b^flox/flox^* controls (Figure 8A). We then assessed the relationship among KIF13B, LRP1, and PPARγ during adipocyte differentiation. In input lysates, KIF13B, LRP1, and PPARγ protein levels were higher in differentiated adipocytes than in SVFs (Figure 8B). Coimmunoprecipitation assays further showed that KIF13B, LRP1, and PPARγ could be detected in the same protein complexes in both SVF cells and differentiated adipocytes, with stronger signals observed after adipogenic differentiation (Figure 8B).

To test whether LRP1 contributes to KIF13B‐induced PPARγ activation, we performed KIF13B overexpression with or without LRP1 knockdown. Subcellular fractionation showed that KIF13B overexpression increased PPARγ abundance, including in the nuclear fraction, whereas LRP1 knockdown blunt this effect (Figure 8C). Consistently, KIF13B overexpression increased PPARγ‐responsive luciferase reporter activity, and this increase was largely abolished by LRP1 knockdown (Figure 8D). At the transcriptional level, KIF13B overexpression upregulated multiple adipogenic and lipid metabolism‐related genes, including *Angptl4*, *Lpl*, *Adipoq*, *Aqp7*, *Plin2*, *Plin1*, *Fabp1*, *Fabp4*, and *Acadm*. The induction of many of these genes was attenuated when LRP1 was silenced (Figure 8E). In line with these molecular changes, oil red O staining showed that KIF13B overexpression increased lipid accumulation during adipocyte differentiation, which could be blocked by LRP1 knockdown (Figure 8F). Given the established link between adipocyte dysfunction, obesity, and T2DM, often involving PPARγ dysregulation [31, 32, 33, 34], we further examined the clinical relevance of this relationship using human adipose single‐cell RNA‐seq datasets. In mature adipocytes from datasets including lean and obese individuals, as well as nondiabetic and diabetic individuals, KIF13B, LRP1, and PPARγ mRNA levels showed positive pairwise correlations (Figure 8G). These associations are consistent with coordinated regulation of this pathway in human adipocytes.

Together, these data suggest that KIF13B promotes PPARγ‐responsive transcriptional activity and adipogenic lipid accumulation in a LRP1‐depedent manner. Based on these findings, we propose a working model in which KIF13B facilitates LRP1‐associated activation of PPARγ transcriptional programs during adipocyte differentiation (Figure 8H).

## 3 Discussion

Currently, most interventions for obesity focus primarily on reducing energy intake and increasing EE through physical activity. However, reducing energy intake often triggers a compensatory decrease in EE by the body, frequently leading to weight regain [35, 36]. Thus, promoting EE may play a more critical role in long‐term weight management than appetite suppression does. In our study, we identifies adipocyte KIF13B as a previously unrecognized regulator of adipose tissue remodeling and obesity‐associated metabolic dysfunction. KIF13B expression was increased in adipose tissue from obese humans and mice and rose during adipogenic differentiation. Genetic deletion of *Kif13b* in adipocytes enhanced thermogenesis and protected mice against HFD‐induced adipose expansion, systemic inflammation, dyslipidemia, insulin resistance, and hepatic steatosis without increasing food intake. In established obesity, AAV9‐mediated adipocyte‐targeted KIF13B knockdown partially reversed metabolic injury, with more pronounced effects in DIO mice than in *ob/ob* mice. Mechanistically, our data verify that KIF13B promotes LRP1‐mediated PPARγ‐responsive adipogenic transcriptional programs. Together, these findings suggest that adipocyte KIF13B is not merely a marker of adipocyte differentiation, but a functional amplifier of pathological adipose tissue expansion under obese conditions.

A key implication of our findings is that KIF13B exerts tissue‐dependent metabolic functions. Our previous study showed that whole‐body or hepatocyte‐specific *Kif13b* deletion aggravates hepatic steatosis and lipid metabolic dysfunction [19]. In contrast, the present study demonstrates that adipocyte‐specific *Kif13b* deletion improves systemic lipid homeostasis and alleviates hepatic steatosis under HFD challenge. Because *Kif13b* was selectively deleted or knocked down in adipocytes rather than hepatocytes, the accompanying reduction in hepatic lipogenesis, lipid uptake‐related genes, and inflammatory markers is unlikely to represent a direct liver‐autonomous effect. A more plausible explanation is adipose‐liver metabolic crosstalk: suppression of adipocyte KIF13B may limit pathological adipose expansion and inflammatory stress, improve peripheral TG handling, reduce circulating lipid burden, and thereby decrease lipid overflow to the liver. The precise mediators remain unresolved. Altered fatty acid flux, adipokine secretion, inflammatory mediators, and other endocrine signals may contribute, and future work using isotope tracing, adipose‐ or liver‐specific rescue models, and direct comparison across tissue‐specific KIF13B models will be required to define this interorgan communication route.

The differential response to adipose KIF13B knockdown in DIO and *ob/ob* mice further indicates that the therapeutic effect of this pathway is context‐dependent. In DIO mice, obesity is driven by chronic dietary nutrient overload and acquired adipose tissue dysfunction; under this condition, inhibition of the KIF13B–LRP1–PPARγ axis reduced adipose inflammation, improved lipid and insulin‐related parameters, and attenuated hepatic steatosis. In *ob/ob* mice, congenital leptin deficiency produces severe hyperphagia, impaired central regulation of energy balance, and suppressed thermogenic tone. This dominant upstream obesogenic drive may limit the ability of adipocyte KIF13B knockdown to reduce body weight or broadly remodel systemic energy metabolism, although inflammatory markers and insulin tolerance were still improved. These findings suggest that KIF13B inhibition may have a broader therapeutic window in acquired obesity driven by nutrient overload than in monogenic obesity dominated by central leptin deficiency.

Previous studies have shown that kinesin family members and other motor proteins regulate vesicle trafficking, exosome‐related transport, and mitochondrial transfer [37, 38]. In our published work, KIF13B was found to promote mitochondrial oxidative phosphorylation by enhancing AMPKα1 phosphorylation [19]. In the present study, adipocyte‐specific *Kif13b* deletion increased UCP1 staining in white and BATs and enhanced mitochondrial oxygen consumption in SVF‐derived adipocytes, supporting improved adipocyte oxidative metabolism, suggesting that the metabolic function of KIF13B may be highly tissue context‐dependent. Unexpectedly, although body weight‐normalized VO_2_, VCO_2_, and EE were increased in *Kif13b^AKO^* mice, analyses of raw metabolic parameters did not show a consistent elevation. Importantly, ANCOVA analysis of raw VO_2_, VCO_2_, and EE using body weight as a covariate did not identify a significant genotype effect. Therefore, the current data support enhanced adipocyte mitochondrial metabolism and thermogenic marker expression, but do not provide definitive evidence for a body weight‐independent increase in whole‐body oxygen consumption or EE.

The improvement in glucose homeostasis after adipocyte *Kif13b* deletion is likely to reflect integrated adipose tissue remodeling rather than a direct effect on canonical insulin signaling alone. In HFD‐fed *Kif13b^AKO^* mice, improved glucose tolerance and insulin responsiveness were accompanied by reduced adiposity, lower circulating lipids, and inflammatory cytokines, decreased adipose macrophage accumulation, and increased UCP1‐positive remodeling. In AAV9‐treated DIO and *ob/ob* mice, adipose *Kif13b* knockdown consistently improved insulin tolerance and reduced inflammatory markers, whereas glucose tolerance and body weight were not uniformly altered. These findings suggest that the insulin‐sensitizing phenotype may be related to reduced adipose inflammation, improved lipid handling, and thermogenic remodeling. Nevertheless, this study did not directly examine canonical insulin signaling events, such as insulin receptor substrate 1 (IRS1) or AKT phosphorylation, in adipose tissue, liver, or skeletal muscle. Building on these findings, we will further investigate how adipocyte KIF13B deficiency influences tissue‐specific insulin signaling and will dissect the relative contributions of adipose inflammation, lipid flux, thermogenesis, and adipose‐derived endocrine signals to the improvement of systemic insulin sensitivity in our future study.

LRP1 is a multifunctional lipoprotein receptor involved in lipid endocytosis, signaling integration, adipogenesis, and systemic energy balance [21, 22, 23, 24, 39, 40, 41]. Previous studies have also suggested that KIF13B can regulate LRP1 localization in other cell types and that LRP1 can interact with PPARγ as a transcriptional coregulatory protein in endothelial cells [18, 25, 42], In the present study, LRP1 signal was reduced in *Kif13b^AKO^* adipose tissue; KIF13B, LRP1, and PPARγ protein levels increased during adipogenic differentiation; and coimmunoprecipitation detected these proteins in the same protein complexes. Functionally, LRP1 knockdown blunted KIF13B‐induced increases in PPARγ abundance, nuclear PPARγ signal, PPARγ‐responsive reporter activity, PPARγ target gene expression, and adipogenic lipid accumulation. Human adipocyte single‐cell datasets further showed positive correlations among KIF13B, LRP1, and PPARγ transcripts. Thus, our data support the interpretation that KIF13B maintains LRP1 abundance and localization and that LRP1 is required for KIF13B‐mediated amplification of PPARγ‐responsive adipogenic programs. These data support a KIF13B–LRP1–PPARγ regulatory axis in adipocytes. However, whether KIF13B‐mediated cytoplasmic transport of LRP1 or establishes that LRP1 functions as a transcriptional coactivator in adipocytes will need live‐cell trafficking analyses, nuclear LRP1 loss‐of‐function experiments, chromatin‐based assays, and domain‐mapping studies to clarify in future studies.

These findings also distinguish KIF13B inhibition from direct systemic targeting of PPARγ. PPARγ is essential for adipocyte differentiation and insulin sensitivity, and global PPARγ agonists such as thiazolidinediones improve glycemic control but are limited by adverse effects including weight gain, edema, and other safety concerns [29, 33, 43, 44]. Our data suggest that KIF13B inhibition attenuates an adipocyte‐enriched LRP1‐dependent amplification arm of PPARγ signaling, thereby reducing pathological adipogenic lipid accumulation while preserving the possibility of basal PPARγ function. However, the present study did not directly compare KIF13B inhibition with adipocyte‐specific *Pparγ* deletion or pharmacological PPARγ modulation. Future side‐by‐side studies will be needed to determine whether KIF13B targeting offers a more selective therapeutic index than direct manipulation of PPARγ or LRP1.

It is worth noting that while we have established KIF13B's role as a molecular motor that spatiotemporally regulates PPARγ activation through LRP1, the upstream events leading to its overexpression and promoter hypomethylation in obesity remain largely unknown. Our previous study found increased methylation of KIF13B in atherosclerotic plaques [45]. It remains unclear whether the elevated expression of KIF13B in obese adipose tissue is related to reduced methylation modification. Elucidating whether specific metabolic signals, alterations in DNMT/TET enzyme activities, or fluctuations in metabolites such as SAM [46, 47] underlie this epigenetic dysregulation represents a critical future direction. Unraveling these initiating mechanisms will not only complete the pathogenic cascade but also provide novel targets for intervening in KIF13B‐mediated adipogenic signaling. Furthermore, although AAV9‐*Adipoq‐shKif13b* effectively reduced adipose KIF13B expression and improved selected metabolic phenotypes, AAV‐based gene delivery has translational limitations, including immunogenicity, dose constraints, and tissue‐targeting issues. Development of adipose‐targeted oligonucleotides, nanoparticles, or small‐molecule inhibitors will therefore be necessary for therapeutic translation. Moreover, in our in vitro experiments, primary mouse SVF‐derived adipocytes were used at Passage 0 and cultured under matched conditions to reduce culture‐induced variation, but MSCs derived from induced pluripotent stem cells (iPSC‐MSCs) have been proposed as an alternative resource to model metabolic disorders in vitro, as they can reduce batch‐to‐batch variation [48, 49]. Thus, future studies using standardized iPSC‐MSC‐derived adipocytes will be valuable to further validate the KIF13B/LRP1/PPARγ pathway and its relationship with inflammatory secretomes, thereby advancing therapeutic development and clinical translation for obesity and metabolic disorders based on this pathway.

It is important to acknowledge the limitations of this study. First, although KIF13B expression was increased in adipose tissue from both individuals and experimental murine models with obesity, the precise mechanism responsible for this change remains unclear. Second, although loss‐ and gain‐of‐function experiments support an LRP1‐dependent effect of KIF13B on PPARγ‐responsive transcription, how KIF13B regulates LRP1 abundance also needs to be investigated in the future. Finally, because obesity profoundly affects cardiovascular homeostasis, adipocyte‐specific *Kif13b* deletion may confer cardiovascular protection through broader interorgan communication involving altered lipid flux, adipokines, or inflammatory mediators. Cardiovascular disease models combined with adipocyte‐specific *Kif13b* manipulation and systematic analysis of adipose–vascular communication will therefore be considered. Addressing these questions will provide a more comprehensive understanding of adipocyte KIF13B in obesity‐associated metabolic and cardiovascular diseases.

## 4 Conclusion

In summary, this study reveals that adipocyte KIF13B promotes obesity‐associated adipose tissue dysfunction by supporting an LRP1‐dependent amplification of PPARγ‐responsive adipogenic programs. Genetic deletion or therapeutic knockdown of adipocyte *Kif13b* limits adipose expansion and inflammation, improves lipid and insulin‐related metabolic parameters, and attenuates hepatic steatosis, particularly in diet‐induced pathological expansion of adipose tissue. These findings connect motor protein‐associated intracellular regulation with nuclear receptor‐dependent adipocyte remodeling and nominate KIF13B as a potential adipose‐focused therapeutic target for obesity‐associated metabolic disorders.

## 5 Materials and Methods

### 5.1 Human Participants

Omentum adipose tissue samples were obtained from normal (*n* = 6) and obese (*n* = 6) individuals who had opted for bariatric or gallbladder surgery and were recruited from the Department of General Surgery at Peking University Third Hospital. Table S1 shows the baseline clinical characteristics of the recruited patients. A detailed medical history was collected from each patient to determine the presence of other cardiovascular disease risk factors (e.g., hypertension, dyslipidemia), the presence of albuminuria, and current medications. Patients were excluded if they had a history of Type 1 diabetes, malabsorption syndrome, Crohn's disease, ulcerative colitis, pancreatitis, or corticosteroid use. VATs from the abdominal region were excised at the end of surgery, and no additional surgical procedures were needed. The tissues were immediately snap‐frozen in liquid nitrogen for subsequent experiments. All patients provided written informed consent. Ethics approval was obtained from the Peking University Third Hospital Medical Science Research Ethics Committee (protocol No. M2024238).

### 5.2 Animal Models

All animal experiments were approved and conducted in strict accordance with the guidelines of the Institutional Animal Care and Use Committee of Peking University (protocol No. LA2023460). The procedures were performed according to the principles outlined in the NIH Guide for the Care and Use of Laboratory Animals (NIH Publication No. 85‐23, revised 1996). WT and *leptin*‐deficient (*ob/ob*) mice were obtained from GemPharmatech (Nanjing, China). Starting at 8 weeks of age, the animals were fed a normal chow diet or a HFD (Research Diet, D12492), as specified in the experimental design. All experimental animals were bred in a specific pathogen‐free (SPF) animal facility with controlled temperature and humidity, a 12‐h light–dark cycle, and free access to food and water at will. The mice were then anesthetized, and the adipose tissues and livers were harvested.

### 5.3 Statistics Analysis

The data are presented as the means ± standard errors of the means (SEMs). Statistical analyses were performed via GraphPad Prism 10. For comparisons between two groups, the normality of the data distribution was assessed via the Shapiro–Wilk test, and variance homogeneity was evaluated via the Brown–Forsythe test. Datasets with a normal distribution and equal variances were analyzed via unpaired Student's *t*‐test, whereas non‐normally distributed data were analyzed via the Wilcoxon rank‐sum test. One‐way analysis of variance (ANOVA) with Tukey's multiple range test was used for experiments with ≥ 3 groups. Two‐way ANOVA with Tukey's multiple range test was applied for analyses with two independent variables. Statistical significance was set at *p* < 0.05.

## Author Contributions

Conceptualization: **Xunde Xian, Guolin Miao**; Methodology: **Guolin Miao, Zhaoling Li, Lianxin Zhang**; Investigation: **Guolin Miao, Zhaoling Li, Lianxin Zhang, Yitong Xu, Liwen Zheng, Ziwei Wang, Aiden Xian, Yufei Han, Jingxuan Chen, Kaikai Lu**; Human VAT sample collection: **Ming Tao, Chunhua Shan, Rui Wei**; Data analysis: **Guolin Miao, Zhaoling Li, Lianxin Zhang**; Bioinformatics analysis: **Zixun Wang**; Visualization: **Guolin Miao, Zhaoling Li, Yitong Xu**; Reagents provision: **Ling Zhang**; Funding acquisition: **Xunde Xian, Erdan Dong, Guolin Miao**; Project administration: **Xunde Xian, Erdan Dong, Wei Huang, Yuhui Wang, Weiguang Zhang**; Supervision: **Xunde Xian, Erdan Dong**; Writing – original draft: **Guolin Miao, Zhaoling Li**; Writing – review and editing: **Xunde Xian**. All the authors have read and approved the article.

## Funding

This work was supported by the National Natural Science Foundation of China (NSFC) 82270479, U25C2004, and HY2021‐1 to Xunde Xian; the Beijing Natural Science Foundation 7242084 to Xunde Xian; the Fundamental Research Funds for the Central Universities to Xunde Xian; Peking University Medicine plus X Pilot Program‐Platform Construction Project 2024YXXLHPT010 to Xunde Xian; the CAMS Innovation Fund for Medical Sciences 2021‐I2M‐5‐003 to Erdan Dong; and the NSFC 82401006 to Guolin Miao.

## Ethics Statement

All animal experiments were approved and conducted in strict accordance with the guidelines of the Institutional Animal Care and Use Committee of Peking University (protocol No. LA2023460). All patients provided written informed consent. Ethics approval was obtained from Peking University Third Hospital Medical Science Research Ethics Committee (protocol No. M2024238).

## Conflicts of Interest

The authors declare no conflicts of interest.

## Supporting information




**Supporting Information File: 1** mco271003‐sup‐0001‐SuppMat.docx

## Data Availability

The RNA‐sequencing data generated in this study have been deposited in the National Center for Biotechnology Information (NCBI) BioProject database under accession number PRJNA1310837. Human visceral and subcutaneous adipose tissue single‐cell RNA‐sequencing data were obtained from the published Cell Metabolism study [50]. Publicly available single‐cell RNA‐sequencing datasets were obtained from the Gene Expression Omnibus database under accession numbers GSE295708, GSE176171, and GSE129363.
