# Supplementary material for: Adipocyte‐Derived KIF13B Aggravates Obesity‐Associated Metabolic Dysfunction via LRP1/PPARγ Signaling
Source: MedComm (2020). 2026 Sep 16;7(10):e71003. doi: 10.1002/mco2.71003 (PMC13583444; doi:10.1002/mco2.71003)
Supplement: Supplementary file 1 [file MCO2-7-e71003-s001.docx]

**Adipocyte-derived KIF13B aggravates obesity-associated metabolic dysfunction via LRP1/PPARγ signaling**

Guolin Miao^1,2,3#^, Zhaoling Li^1,#^, Lianxin Zhang^1,#^, Yitong Xu^1,#^, Ziwei Wang^4,#^, Zixun Wang^1^, Aiden Xian^5^, Liwen Zheng^1^, Jingxuan Chen^1^, Yufei Han^1^, Chunhua Shan^6^, Kaikai Lu^1^, Ming Tao^7^, Ling Zhang^1^, Weiguang Zhang^8^, Wei Huang^1^, Yuhui Wang^1^, Rui Wei^6^, Erdan Dong^1,2,9^, Xunde Xian^1,8,10*^.

**Affiliations**

^1^ Institute of Cardiovascular Sciences, State Key Laboratory of Vascular Homeostasis and Remodeling, School of Basic Medical Sciences, Peking University, Beijing, China.

^2^ Department of Cardiology and Institute of Vascular Medicine, Peking University Third Hospital, Beijing, China.

^3^ Beijing Institute of Heart, Lung and Blood Vessel Diseases, Beijing Anzhen Hospital, Capital Medical University, Beijing, China.

^4^ Department of Endocrinology, Peking University First Hospital, Beijing, China.

^5^ Shepton High School, Plano, Texas, United States.

^6^ Department of Endocrinology and Metabolism, State Key Laboratory of Female Fertility Promotion, Peking University Third Hospital, Beijing, China

^7^ Department of General Surgery, Peking University Third Hospital, Beijing, China

^8^ Department of Human Anatomy and Histology and Embryology, School of Basic Medical Sciences, Peking University, Beijing, China

^9^ Research Unit of Medical Science Research Management/Basic and Clinical Research of Metabolic Cardiovascular Diseases, Chinese Academy of Medical Sciences, Beijing, China

^10^ Beijing Key Laboratory of Cardiovascular Receptors Research, Peking University Third Hospital, Beijing, China

*Corresponding author: Xunde Xian, PhD, Institute of Cardiovascular Sciences, State Key Laboratory of Vascular Homeostasis and Remodeling, School of Basic Medical Sciences, Peking University, No. 38 Xueyuan Rd, Haidian District, Beijing, China 100191. E-mail: xianxunde@bjmu.edu.cn.

^#^G.M., Z.L., L.Z., Y.X. and Z.W. contributed equally to this article.

**Supplementary Materials and Methods**

**1. Generation and Genotyping of *Kif13b^AKO^* Mice**

*Kif13b*-*floxed* (*Kif13b*^flox/flox^) mice were generated on a C57BL/6J background using CRISPR/Cas9-mediated genome editing at the Institute of Laboratory Animal Science, Chinese Academy of Medical Sciences & Peking Union Medical College (Beijing, China) ^1^. The donor template was designed on the basis of the mouse genomic sequence (GRCm39), featuring two loxP sites flanking exon 6. Zygotes were microinjected with a mixture of Cas9 protein and sgRNAs, with or without the donor template. Adipose tissue-specific knockout mice (*Adipoq-Cre;Kif13b^flox/flox^*, hereafter referred to as *Kif13b^AKO^*) were subsequently generated by crossing *Kif13b*^flox/flox^ mice with *Adipo-Cre* transgenic mice on a C57BL/6J background.

*Kif13b^flox/flox^* and *Kif13b^AKO^* mice were genotyped via tail genomic DNA sequencing, and Southern blot examination was performed to further confirm the correct genotype with an Easy Pure Genomic DNA Kit. The sequences of primers used for genotype identification are presented in Table S3 for reference.

**2. Glucose and Insulin Tolerance Tests**

Glucose tolerance tests (GTTs) were performed on the mice after a 12-hour fast. The mice received an intraperitoneal injection of 20% glucose solution (10 ml/kg body weight), and blood glucose levels were measured at 0, 15, 30, 60, 90, and 120 minutes using a glucometer (Bayer Contour). For the insulin tolerance test (ITT), the mice were fasted for 6 hours prior to the assay. Following the determination of basal glucose concentrations, each mouse was injected intraperitoneally with insulin (1 IU/kg), and glucose levels were assessed at 15, 30, 60, 90, and 120 minutes post-injection.

**3. AAV9-Mediated Knockdown of KIF13B**

WT and *ob/ob* mice were injected with AAV9-*Adipoq*-*shKif13b* or AAV9-*Adipoq*-*scramble*) at a dose of 2*10^11 vg/mouse via the tail vein. The total injection volume was 500 µl, as the AAV9 stock solution was further diluted with saline. The *shKif13b* sequence was present in Table S2.

**4. Histology and Immunochemistry**

For histological analysis, liver and adipose tissues were fixed in 4% polyoxymethylene, paraffin-embedded, sectioned at 5 μm, and stained with hematoxylin and eosin (H&E) or immunostained with antibodies against UCP1 (ABclonal, A5857, 1:100) and CD68 (Boster, BM3639, 1:100). Adipocyte size was quantified using Fiji: ImageJ on 100 randomly selected fields per mouse and the results are presented as a frequency distribution histogram of cell diameters. UCP1- or CD68-positive area and crown-like structures were quantified in a blinded manner under the same imaging and thresholding conditions.

**5. Immunofluorescence Staining**

Antigen retrieval was performed on adipose tissue paraffin sections after dewaxing and rehydration. Following blocking with PBS containing 3% (w/v) bovine serum albumin (BSA), the sections were incubated overnight at 4 °C with LRP1 (MCE, HY-P80746, 1:100) or KIF13B (Sigma-Aldrich, SAB2101257, 1:100) antibodies in blocking buffer. After being washed with PBS containing 0.1% (v/v) Tween 20, the sections were incubated with Alexa Fluor-conjugated secondary antibodies at a 1:1000 dilution for 1 h at 37 °C in the dark. Nuclei and lipid droplets were visualized using 4',6-diamidino-2-phenylindole (DAPI).

**6. Hepatic lipid measurements**

20 mg of liver tissue was homogenized in 1ml cold phosphate buffer solution (PBS), and then 4 ml of chloroform/methanol (v:v = 2:1) was added. The mixture was vortexed for 2 min, and then allowed to stand for 20 min. After a centrifugation at 3000 rpm for 30 min, the chloroform layer was transferred to a new glass tube using a glass syringe and dried under a nitrogen stream. Lipids were dissolved with 500 μl of 3% triton X-100, and the contents of cholesterol and triglyceride were measured by TC (BioSino, 100000180) and TG (BioSino, 100000220) kits according to the manufacturer’s protocol.

**7. Energy Metabolism Characterization**

Whole-body energy metabolism was assessed by indirect calorimetry using a CLAMS-HC system equipped with an Oxymax open-circuit indirect calorimetry module (Oxymax v5.52, Columbus Instruments). Mice were individually housed in metabolic chambers with free access to food and water. Before data acquisition, mice were acclimated to the metabolic chambers for 2 days at 22 °C, and data collected during this acclimation period were excluded from the analysis. Metabolic parameters were then recorded continuously for 24 h on day 3.

The CLAMS-HC system recorded oxygen consumption (VO_2_), carbon dioxide production (VCO_2_), respiratory exchange ratio (RER), energy expenditure, food intake and locomotor activity. VO_2_, VCO_2_ and energy expenditure were exported from the Oxymax software as body weight-normalized values and are presented as ml/kg/hour for VO_2_ and VCO_2_, and kcal/kg/hour for energy expenditure. RER was calculated as VCO_2_/VO_2_ and was analyzed as a dimensionless parameter. Locomotor activity was recorded as total infrared beam breaks in the X–Y plane with 1-inch beam spacing.

Metabolic cage data were analyzed on a per-animal basis. For each mouse, values were averaged over the 24-hour recording period or analyzed as area under the curve (AUC), as indicated in the Figure legends. Body weight-normalized VO_2_, VCO_2_, energy expenditure and RER were first analyzed using the values directly exported from the Oxymax software. To evaluate whether genotype-associated differences in indirect calorimetry parameters were independent of body weight, absolute per-mouse VO_2_, VCO_2_ and energy expenditure values were reconstructed from the exported body weight-normalized values by multiplying each value by the corresponding body weight in kilograms. Because RER is a ratio and is not normalized to body weight, RER values were analyzed directly without body weight conversion.

Absolute VO_2_, VCO_2_ and energy expenditure values were further analyzed using an ANCOVA-like multiple linear regression model in GraphPad Prism 10.0. In these models, genotyping was included as a categorical independent variable, and body weight was included as a continuous covariate. Each model included an intercept and the main effects of genotype and body weight. Adjusted genotype effects were reported as regression coefficients with 95% confidence intervals, F values and P values. Model diagnostics, including residual distribution and multicollinearity, were assessed using Prism diagnostic outputs.

**8. Body composition analysis**

Total fat mass was determined using a Minispec mq7.5 series nuclear magnetic resonance (NMR) analyzer (Bruker, France). Briefly, conscious mice were individually placed in the measurement chamber, and whole-body fat mass was measured according to the manufacturer’s instructions. Total fat mass values were recorded for subsequent analysis.

**9. Coimmunoprecipitation (Co-IP) Assay**

Cultured cells were lysed in 1 ml of RIPA buffer on ice for 30 min, vortexed for 15 s, and centrifuged at 12,000 × g for 30 min at 4 °C. Eighty microliters of the supernatant were mixed with 20 μl of 5× loading buffer as input samples. The remaining 800 μl of the supernatant was incubated with 20 μl of LRP1 (MCE, HY-P80746), PPARγ (Proteintech, 66936-1-Ig) or KIF13B (Sigma-Aldrich, SAB2101257) antibody at 4 °C for 6 h, followed by overnight incubation with protein A/G agarose beads. The beads were washed 5 times with lysis buffer at 4 °C, boiled with 120 μl of 1× loading buffer at 95 °C for 10 min, and centrifuged at 12,000 × g for 5 min. The supernatants were used as IP samples for immunoblotting.

**10. RNA isolation and mRNA analysis**

Total RNA was extracted from cells/adipose tissue via TRIzol Reagent Kit (Invitrogen, 15596018). Reverse transcription was performed with 5 μg of total RNA via a First-Strand cDNA Synthesis Kit (TransGen, AT301-03). Real-time quantitative polymerase chain reaction (qPCR) was carried out using a SYBR PCR kit (TransGen, AQ132-24) with QuantStudio 3 (Thermo Fisher) in accordance with the manufacturers’ instructions. *Gapdh* was used as internal controls for the qPCR. The qPCR primer sequences are present in Table S4.

**11. Cell Culture and Induction of Differentiation**

The epididymal fat pads of 6-week-old male mice were dissected and digested for 1 h at 37 °C with collagenase II in DMEM with mechanical agitation. The same volume of DMEM containing 1% BSA was added to terminate the digestion. The digests were filtered through a 70-μm cell strainer, and the cell suspensions were centrifuged at room temperature and 500 × g for 20 min. Floating mature adipocytes were collected separately, while the stromal vascular fraction (SVF) cells were plated in 24- or 12- or 6-well plates and maintained with DMEM/F12 medium supplemented with 10% fetal bovine serum (FBS, TransGen Biotech, FS401-02) and 1% penicillin-streptomycin (P/S, TransGen Biotech, FG101-01) at 37 °C in a humidified 5% CO_2_ incubator. When the cells reached 95% confluence, differentiation was induced by changing the medium to induction medium (DMEM/F12 containing 10% FBS, 1% P/S, 10 μg/mL insulin (MCE, HY-P0035A), 0.5 mM 3-isobutyl-1-methylxanthine (MCE, [HY-12318](https://www.medchemexpress.cn/IBMX.html)), 1 μM dexamethasone (MCE, HY-14648), and 2.5 μM rosiglitazone (MCE, HY-17386)) and incubated for 2 days. Then, the cells were switched to maintenance medium (DMEM/F12 containing 10% FBS, 1% P/S, and 10 μg/mL insulin) for 2 days, followed by replacement with fresh maintenance medium and incubation for another 2 days.

**12. Measurement of cellular oxygen consumption rate (OCR)**

The oxygen consumption rate (OCR) of adipocytes was measured using an XFe96 Extracellular Flux Analyzer (Seahorse Bioscience, USA) according to the manufacturer’s instructions. Briefly, stromal vascular fraction (SVF) cells were isolated from *Kif13b^flox/flox^* and *Kif13b^AKO^* mice and seeded into Seahorse XF96 cell culture microplates at a density of 5 × 10^4 cells per well. Cells were induced to differentiate into adipocytes for 7 days in induction medium.

Before the assay, differentiated adipocytes were incubated for 1 h at 37 °C in a non-CO₂ incubator in Seahorse assay medium consisting of XF base medium supplemented with 2 mM glutamine, 1 mM sodium pyruvate and 25 mM glucose. OCR was measured under basal conditions and after sequential injection of oligomycin (1.5 μM), carbonyl cyanide 4-(trifluoromethoxy) phenylhydrazone (FCCP, 1.5 μM), and rotenone/antimycin A (1.5 μM). Three measurement cycles were recorded for each condition. Basal respiration, ATP-linked respiration and maximal respiration were calculated from the OCR profile.

**13. Transfection of Plasmids**

Plasmids carrying *KIF13B* or null vectors were purchased from Sangon Biotech (China, Shanghai) and were transduced into SVFs via the liposomal transfection reagent (Yeasen, 40802ES03) following the manufacturer’s protocol. The cells were seeded in 6-well plates and reached 80–85% confluence at the time of transfection. After 6 h of transfection, the Opti-MEM medium (Gibco, 31985070) was changed, and fresh DMEM/F12 plus 1% P/S was added. Finally, the cells were placed on ice, washed with ice-cold PBS, and collected for qPCR and Western blotting.

**14. RNA Interference**

Preadipocytes were transfected with *siLRP1* or *scramble* via Lipofectamine RNA iMAX (Invitrogen, 13778150) as described by the manufacturer. The cells were seeded in 6-well plates and reached 80–90% confluence at the time of transfection. After 6 h of transfection, the Opti-MEM was replaced with serum-free DMEM/F12. Finally, the cells were placed on ice, washed with ice-cold PBS, and collected for qPCR and Western blotting assays.

**15. Luciferase Reporter Assay**

The PPARγ luciferase reporter plasmid based on the PPRE sequence and the pRL-TK vector, which was used as an external control, were transfected into SVFs with liposomal transfection reagent (Yeasen, 40802ES03) following the manufacturer’s protocol. After 48 h of transfection, the cells were induced to differentiate into adipocytes. Adipocytes were lysed with passive lysis buffer, and the luciferase activity of the sample was measured with a dual luciferase assay system (Yeasen, 11402ES60) in accordance with the manufacturer’s protocol. The final data for each sample were calculated by normalizing the PPARγ reporter’s luciferase value to the pRL-TK vector’s luciferase value.

**16. Biochemical Analysis**

After a 4-h fast, blood was collected from the retro-orbital venous plexus into heparinized tubes, and plasma was isolated by centrifugation at 4 °C and 4,000 rpm for 10 min. Plasma total cholesterol (TC, BioSino, 100000180), triglyceride (TG, BioSino, 100000220), glucose (BioSino, 100000240), insulin (FUJIFILM Wako, 638-01489), nonesterified fatty acid (NEFA, FUJIFILM Wako, 633-52001), alanine aminotransferase (ALT, Nanjing Jiancheng Bioengineering Institute, C009-2-1), aspartate aminotransferase (AST, Nanjing Jiancheng Bioengineering Institute, C010-2-1), interleukin (IL)-1β (Ruixinbio, RXW203063M), IL-6 (Ruixinbio, RXW203049M), and C-C motif chemokine ligand 5 (CCL5, Abcam, Ab100739) levels were measured via commercial kits according to the manufacturers’ instructions.

**17. Protein extraction and western blotting**

Tissues or cells were harvested and lysed in RIPA buffer (APPLYGEN, C1053-500) supplemented with protease inhibitor (Roche, 04693132001) and phosphatase inhibitor (Roche, 4906845001). Lysates were placed on ice and vortexed every 10 min for half an hour before centrifugation for 30 min at 12,000 g, 4 °C. The supernatants were collected and protein concentration was measured by Pierce BCA Protein Assay (Thermo Fisher, 23225). Protein lysates were heated in 1X loading buffer at 37 °C for 1 h. Proteins were loaded in SDS-PAGE, transferred to nitrocellulose (NC) membrane, and blocked in skim milk for 1 h before incubating with the indicated primary antibodies overnight. After washing with 1X TBST three times (10 min each time), the second antibodies were added for incubation for another 1 h. Finally, after another three washes with 1X TBST, the protein bands were detected with ECL Kit (Solarbio, PE0010) by iBright 1500 (Invitrogen).

**18. Plasma LPL activity**

Eight-week-old *Kif13b*^flox/flox^ or *Kif13b*^AKO^ mice were fed an HFD for 8 weeks and fasted overnight before analysis. Mice were intraperitoneally injected with heparin at 2 U/g body weight. Blood samples were collected 30 min after injection and immediately centrifuged at 4,000 rpm for 10 min to isolate plasma. Plasma LPL activity was measured using an LPL Activity Kit (Abcam, ab204721) following the manufacturer’s procedures with minor modifications.

**19. Oral fat load test**

Eight-week-old *Kif13b*^flox/flox^ or *Kif13b*^AKO^ mice were fed an HFD for 8 weeks and starved for 12 h and weighed before fasting blood was taken. Then these mice were gavaged with olive oil (10ul/g, La Sicilia, 8011845004871), and blood was collected from the intra-orbital vein accordingly, at 30 min, 1 h, 2 h, 4 h, 6h and 8 h after the olive oil challenge.

**20. Oil red O staining**

Mice were anesthetized by inhalation of carbon dioxide. Livers were harvested, fixed with 4% paraformaldehyde for 12-24 h, embedded in OCT (Sakura, 4583), cryosectioned at 7 µm, and stored at -20 °C. Tissue sections were rewarmed at room temperature and washed with distilled water three times. Slides were soaked in 60% isopropanol for 10 s and then stained with 0.3% freshly prepared oil red O solution (Sigma-Aldrich, O0625) for 30 min. 60% isopropanol was used to wash away floating color, and cell nuclei were counterstained with hematoxylin. Next, 90% glycerin was mounted in the center of tissue sections to seal the slides. For cells, SVF-derived adipocytes were fixed in 4% PFA for 10 min prior to being stained with 60% oil red O for 30 min.

**21.RNA-Sequencing Data Analysis**

Total RNA was extracted from **the epididymal white adipose tissues** of *Kif13b^flox/flox^* or *Kif13b^AKO^* mice fed an HFD for 12 weeks. RNA sequencing analysis of **epididymal white adipose** tissues was performed by Shanghai Majorbio Bio-Pharm Technology Co., Ltd. (Shanghai, China). All RNA sequencing data were analyzed using the free online platform of Majorbio Cloud Platform (www.majorbio.com). Differentially expressed genes (DEGs) were identified based on the criteria of Padjust <0.05 and |log2FC| > 1.

**22. Single-cell RNA-Sequencing (scRNA-Seq) analysis**

The Python package scanpy was used to perform upstream scRNA-Seq analysis^2^. After filtering cells and genes, we used the scVI package to remove batch effect and perform data embedding, followed by a manual selection of cell type markers to annotate cell types^3^.

**Figure S1.** **Single-cell transcriptomic analysis of *KIF13B* expression across cell populations in human subcutaneous adipose tissue.** (A) Uniform manifold approximation and projection (UMAP) visualization of major cell populations identified in human subcutaneous adipose tissue from the GSE295708 dataset (n = 6 per group). (B) Violin plots showing *kinesin family member 13B* (*KIF13B*) mRNA levels across adipose-resident cell populations in lean and obese individuals from GSE295708. (C) UMAP visualization of major cell populations identified in human subcutaneous adipose tissue from the GSE176171 dataset (Normal, n = 5; Obese, n = 8). (D) Violin plots showing *KIF13B* mRNA levels across adipose-resident cell populations in normal-weight and obese individuals from GSE176171. (E) UMAP visualization of cell populations in subcutaneous adipose tissue stratified by metabolic status. (F) Violin plots showing *KIF13B* mRNA levels across cell populations in metabolically healthy obese (MHO, n = 32) and metabolically unhealthy obese (MUO, n = 45) individuals. ASPC, adipose stem/progenitor cell; EC, endothelial cell; LEC, lymphatic endothelial cell; SMC, smooth muscle cell; VEC, vascular endothelial cell; MHO, metabolically healthy obesity; MUO, metabolically unhealthy obesity.

**Figure S2.** **Single-cell transcriptomic analysis of *KIF13B* expression across cell populations in human visceral adipose tissue.** (A) UMAP visualization of major cell populations identified in human visceral adipose tissue from the GSE176171 dataset (Normal, n = 5; Obese, n = 8). (B) Violin plots showing *KIF13B* mRNA levels across adipose-resident cell populations in normal-weight and obese individuals from GSE176171. (C) UMAP visualization of visceral adipose tissue cell populations stratified by metabolic status. (D) Violin plots showing *KIF13B* mRNA levels across cell populations in metabolically healthy obese (MHO, n = 32) and metabolically unhealthy obese (MUO, n = 45) individuals. (E) UMAP visualization of major cell populations identified in human visceral adipose tissue from the GSE129363 dataset(n = 6 per group). (F) Violin plots showing *KIF13B* mRNA levels across cell populations in nondiabetic and diabetic individuals from GSE129363. ASPC, adipose stem/progenitor cell; EC, endothelial cell; LEC, lymphatic endothelial cell; SMC, smooth muscle cell; cMesoC, classical mesothelial cell; meMesoC, mesenchymal-like mesothelial cell; MHO, metabolically healthy obesity; MUO, metabolically unhealthy obesity.

**Figure S3.** **Generation and validation of adipocyte-specific *Kif13b* knockout mice**. (A) Schematic representation of the strategy used to generate adipocyte-specific *Kif13b* knockout mice. In *Kif13b^flox/flox^* mice, exon 6 of *Kif13b* was flanked by two loxP sites. Cre recombinase driven by the *Adipoq* promoter mediated adipocyte-specific deletion of the floxed allele, generating *Adipoq-Cre*;*Kif13b^flox/flox^* mice, hereafter referred to as *Kif13b^AKO^* mice. Primer locations and expected amplicon sizes are indicated. (B) qPCR analysis of *Kif13b* mRNA levels in epididymal white adipose tissue (eWAT), brown adipose tissue (BAT), lung, testis, heart, brain, skeletal muscle, liver and jejunum from *Kif13b^flox/flox^* and *Kif13b^AKO^* mice (n = 6 per group). (C) Western blot analysis of KIF13B protein expression in eWAT and BAT from *Kif13b^flox/flox^* and *Kif13b^AKO^* mice. GAPDH was used as a loading control (n = 6 per group). (D) Representative immunofluorescence staining of KIF13B in eWAT from *Kif13b^flox/flox^* and *Kif13b^AKO^* mice. Rabbit immunoglobulin G (IgG) was used as a negative control. KIF13B is shown in red and nuclei were counterstained with 4′,6-diamidino-2-phenylindole (DAPI) in blue. Scale bar, 100 μm. (E) Quantification of relative KIF13B fluorescence intensity in eWAT (n = 7 per group). (F) Representative immunofluorescence staining and quantification of KIF13B expression in inguinal white adipose tissue (iWAT) from *Kif13b^flox/flox^* (n = 7) and *Kif13b^AKO^* (n = 7) mice. Rabbit IgG was used as a negative control. Scale bar, 100 μm. (G) Representative immunofluorescence staining and quantification of KIF13B expression in BAT from *Kif13b^flox/flox^* (n = 7) and *Kif13b^AKO^* (n = 7) mice. Rabbit IgG was used as a negative control. Scale bar, 50 μm.

Data are presented as mean ± standard error of the mean (SEM). In panel (B), multiple unpaired t tests were carried out to determine the *p* value. For panels (E), (F) and (G), *p* values were determined via unpaired Student’s t tests.

**Figure S4.** **KIF13B deficiency enhances mitochondrial oxidative respiration in differentiated adipocytes.** (A) Oxygen consumption rates (OCR) were measured by Seahorse XF96 analyzer in SVFs isolated from *Kif13b^flox/flox^* and *Kif13b^AKO^* mice. SVFs were induced to differentiate into adipocytes with Dulbecco’s modified Eagle medium/Ham’s F-12 (DMEM/F-12) containing 10% fetal bovine serum (FBS), 1% penicillin/streptomycin (P/S), 10 μg/ml insulin, 0.5 mM 3-isobutyl-1-methylxanthine, 1 μM dexamethasone, and 2.5 μM rosiglitazone for 7 days (n = 8 independent biological replicates). (B) OCR relating to mitochondrial basal respiration, maximal respiration, and adenosine triphosphate (ATP) production capacity was respectively analyzed and normalized to the corresponding total protein content per well. n = 8 independent biological replicates.

Data are presented as mean ± SEM. P values in panel (B) was determined by unpaired Student's t test.

**Figure S5. Adipocyte-specific *Kif13b* deletion is associated with higher body weight-normalized metabolic rates during HFD feeding.** (A) Core body temperature in high fat diet (HFD)-fed *Kif13b^flox/flox^* and *Kif13b^AKO^* mice (*Kif13b^flox/flox^*, n = 7; *Kif13b^AKO^*, n = 9). (B-G) Twenty-four-hour indirect calorimetry analysis of food intake (B), oxygen consumption (VO_2_) (C), carbon dioxide production (VCO_2_) (D), respiratory exchange ratio (RER) (E), locomotor activity counts (F) and energy expenditure (EE) (G) in HFD-fed *Kif13b^flox/flox^* and *Kif13b^AKO^* mice for 9 weeks (n = 6 per group). VO_2_, VCO_2_ and EE are presented as body weight-normalized values exported from the Oxymax software and summarized as area under the curve (AUC) where indicated.

Data are presented as mean ± SEM. P values were determined by unpaired Student's t test.

**Figure S6.** **Absolute indirect calorimetry parameters in HFD-fed *Kif13b^AKO^* mice.** (A-E) 24-hour indirect calorimetry analysis of absolute per-mouse VO_2_ (A), absolute per-mouse VCO_2_ (B), RER (C), absolute per-mouse EE (D) and locomotor activity counts (E) during the dark phase, light phase and total recording period in HFD-fed *Kif13b^flox/flox^* and *Kif13b^AKO^* mice (n = 6 per group). Absolute VO2, VCO_2_ and EE values were reconstructed from body weight-normalized Oxymax outputs by multiplying by the corresponding body weight. Body weight-adjusted analysis of covariance (ANCOVA)-like regression analyses of absolute VO_2_, VCO_2_ and EE are summarized in Supplementary Table 5.

Data are presented as mean ± SEM. P values were determined by multiple unpaired t tests.

**Figure S7. Absolute indirect calorimetry parameters in AAV9-*Adipoq-shKif13b*-treated DIO mice.** Eight-week-old male wild-type (WT) mice were fed an HFD for 8 weeks, injected via the tail vein with adeno-associated virus serotype 9 (AAV9)-*Adipoq-scramble* or AAV9-*Adipoq-shKif13b*, and maintained on HFD for an additional 8 weeks. (A-D) 24-hour indirect calorimetry analysis of absolute per-mouse VO_2_ (A), absolute per-mouse VCO_2_ (B), RER (C) and absolute per-mouse EE (D) during the dark phase, light phase and total recording period in diet-induced obesity (DIO) mice treated with AAV9-*Adipoq*-*scramble* or AAV9-*Adipoq-shKif13b* (n = 6 per group). Absolute VO_2_, VCO_2_ and EE values were reconstructed from body weight-normalized Oxymax outputs by multiplying by the corresponding body weight. Body weight-adjusted ANCOVA-like regression analyses of absolute VO_2_, VCO_2_ and EE are summarized in Supplementary Table 6.

Data are presented as mean ± SEM. P values were determined by multiple unpaired t tests.

**Figure S8.** **AAV9-mediated KIF13B knockdown attenuates hepatic triglyceride accumulation in DIO mice.** (A) Liver weight and liver weight normalized to body weight in DIO mice treated with AAV9-*Adipoq-scramble* or AAV9-*Adipoq*-*shKif13b* (n = 6 per group). (B) Plasma alanine aminotransferase (ALT) and aspartate aminotransferase (AST) levels (n = 6 per group). (C) Representative hematoxylin and eosin (H&E) and oil red O staining of liver frozen sections. Scale bars, 100 µm. (D) Hepatic total cholesterol (TC) and triglyceride (TG) contents (n = 6 per group). (E) Relative mRNA levels of hepatic genes related to de novo lipogenesis, lipid uptake and inflammation (n = 6 per group).

Data are presented as mean ± SEM. P values in panels (A), (B) and (D) were determined by unpaired Student's t test. P values in panel (E) were determined by multiple unpaired t tests.

**Figure S9. AAV9-mediated KIF13B knockdown remodels adipose tissue in *ob/ob* mice.** (A) Schematic diagram of AAV9-*Adipoq*-*shKif13b* intervention in *ob/ob* mice. Eight-week-old male *ob/ob* mice were injected via the tail vein with AAV9-*Adipoq*-*scramble* or AAV9-*Adipoq*-*shKif13b* and maintained on a chow diet for 12 weeks. Glucose tolerance test (GTT) was performed at week 4, insulin tolerance test (ITT) at week 7 and mice were sacrificed at week 12. (B) Quantitative polymerase chain reaction (qPCR) analysis of *Kif13b* mRNA levels in eWAT (n = 6 per group). (C, D) Representative KIF13B immunofluorescence staining in adipose tissue (C) and quantification of KIF13B fluorescence intensity (D) (n = 6 per group). Rabbit IgG was used as a negative control. Scale bar, 100 µm. (E) Body weight during the treatment period (n = 6 per group). (F) Weights of major adipose depots, including eWAT, subcutaneous white adipose tissue (sWAT), iWAT and BAT . (G) Representative H&E staining and immunohistochemical staining for cluster of differentiation 68 (CD68) and uncoupling protein 1 (UCP1) in eWAT. Asterisks indicate crown-like structures; black arrows indicate CD68- or UCP1-positive staining. Scale bars, 200 µm. (H) Adipocyte size distribution in eWAT. For each mouse, 500 randomly selected fields of view were analyzed. (I) Quantification of crown-like structures, CD68-positive area and UCP1-positive area in eWAT. (J) Representative H&E staining of BAT. Scale bar, 100 µm. (K) Representative UCP1 immunohistochemical staining and quantification of UCP1-positive area in BAT. Scale bar, 100 µm. (F-H, and K) AAV9-*Adipoq*-*scramble*-treated *ob/ob* mice: n = 6, AAV9-*Adipoq*-*shKif13b*-treated *ob/ob* mice: n = 5.

Data are presented as mean ± SEM. In panels (B), (D), (I) and (K), p values were determined via unpaired Student’s t tests. For panel (E), p values were analyzed by two-way ANOVA followed by Tukey's multiple-comparisons test. For panel (F), multiple unpaired t tests were carried out to determine the p value.

**Figure S10. Systemic metabolic and body weight-normalized indirect calorimetry parameters in AAV9-*Adipoq*-*shKif13b*-treated *ob/ob* mice.** Eight-week-old male *ob/ob* mice were injected via the tail vein with AAV9-*Adipoq*-*scramble* or AAV9-*Adipoq*-*shKif13b* and maintained on a chow diet for 12 weeks. (A) Fasting plasma TC, TG and non-esterified fatty acid (NEFA) levels in *ob/ob* mice treated with AAV9-*Adipoq*-*scramble* or AAV9-*Adipoq*-*shKif13b*. (B) Plasma interleukin-6 (IL-6), interleukin-1β (IL-1β), and C-C motif chemokine ligand 5 (CCL5) levels. (C) Fasting plasma glucose levels. (D) Fasting plasma insulin levels. (E, F) GTT (E) and AUC analysis (F). (G, H) ITT (G) and AUC analysis (H). (A-H) AAV9-*Adipoq-scramble*-treated *ob/ob* mice: n = 6, AAV9-*Adipoq*-*shKif13b*-treated *ob/ob* mice: n = 5. (I-L) 24-hour indirect calorimetry analysis of body weight-normalized VO_2_ (I), VCO_2_ (J), RER (K) and EE (L), with corresponding AUC values where indicated, n = 4 per group. VO_2_, VCO_2_ and EE are presented as body weight-normalized values exported from the Oxymax software.

Data are presented as mean ± SEM. P values for two-group comparisons and AUCs were determined by unpaired Student's t test. GTT and ITT time-course data were analyzed by two-way analysis of variance (ANOVA) followed by Tukey's multiple-comparisons test.

**Figure S11. Absolute indirect calorimetry parameters in AAV9-*Adipoq*-*shKif13b*-treated *ob/ob* mice.** Eight-week-old male *ob/ob* mice were injected via the tail vein with AAV9-*Adipoq*-*scramble* or AAV9-*Adipoq*-*shKif13b* and maintained on a chow diet for 12 weeks. (A-D) 24-hour indirect calorimetry analysis of absolute per-mouse VO_2_ (A), absolute per-mouse VCO_2_ (B), RER (C) and absolute per-mouse EE (D) during the dark phase, light phase and total recording period in *ob/ob* mice treated with AAV9-*Adipoq*-*scramble* or AAV9-*Adipoq*-*shKif13b* (n = 4 per group). Absolute VO_2_, VCO_2_ and EE values were reconstructed from body weight-normalized Oxymax outputs by multiplying by the corresponding body weight. Data are presented as mean ± SEM. P values were determined by multiple unpaired t tests.

**Figure S12. AAV9-mediated KIF13B knockdown attenuates hepatic lipid accumulation in *ob/ob* mice**. Eight-week-old male *ob/ob* mice were injected via the tail vein with AAV9-*Adipoq*-*scramble* or AAV9-*Adipoq*-*shKif13b* and maintained on a chow diet for 12 weeks. AAV9-*Adipoq*-*scramble*-treated *ob/ob* mice: n = 6, AAV9-*Adipoq*-*shKif13b*-treated *ob/ob* mice: n = 5. (A) Liver weight and liver weight normalized to body weight in *ob/ob* mice treated with AAV9-*Adipoq*-*scramble* or AAV9-*Adipoq*-*shKif13b*. (B) Plasma ALT and AST levels. (C) Representative H&E and oil red O staining of liver sections. Scale bars, 100 µm. (D) Hepatic TC and TG contents. (E) Relative mRNA levels of hepatic genes related to de novo lipogenesis, lipid uptake and inflammation. Data are presented as mean ± SEM. P values in panels (A), (B) and (D) were determined by unpaired Student's t test. P values in panel (E) were determined by multiple unpaired t tests.

**Table S1** **Characteristics of human subjects**

|  | Normal | Obese |  |
| --- | --- | --- | --- |
| n | 6 | 6 |  |
| Male:Female (n) | 4:2 | 4:2 |  |
|  | Mean± SEM | Mean± SEM | *p* value |
| Age (years) | 32.83±3.790 | 35.33±2.940 | 0.6136 |
| Body weight (kg) | 68.33±5.352 | 131.6±6.586 | <0.0001 |
| BMI (kg/m^2^) | 22.47±0.9493 | 43.20±2.326 | <0.0001 |

*p* values are calculated used unpaired Student t test.

**Table S2 *shKif13b* sequences**

| **Gene name** | **Species** | **Gene ID** | **Sequences** |
| --- | --- | --- | --- |
| *Kif13b* | *[Mus musculus](https://www.ncbi.nlm.nih.gov/datasets/taxonomy/10090)* | 16554 | 5'-GCAGATAACTATGACGAAACC-3’ |

**Table S3 Mouse *Lrp1* gene siRNA sequences**

| **Gene name** | **Sequences sense (5’** – **3’)** | **Anti-sense (5’** – **3’)** |
| --- | --- | --- |
| si*Lrp1*-1 | AGAUCUGUCCACAGAGUAA | UUACUCUGUGGACAGAUCU |
| si*Lrp1*-2 | CCAUGUCUCGUCUCUGCAA | UUGCAGAGACGAGACAUGG |
| si*Lrp1*-3 | CAAAUCACCUGUAUCUCAA | UUGAGAUACAGGUGAUUUG |

**Table S4 qPCR primer sequences**

| **Mouse primer sequences** | | | |
| --- | --- | --- | --- |
| **Gene name** | **Forward primer** | | **Reverse primer** |
| *Kif13b* | AACGAACCCAGAAAGAGGA | | GCTTGTGACAGCCAGTTTA |
| *Angptl4* | ACTTCCTCCTCGTCTCTG | | TCGTTCCTGCCTCATTATAG |
| *Lpl* | CGCTCCATTCATCTCTTCA | | TACATCTTGCTGCTTCTCTT |
| *Adipoq* | GACGACAGGAAGGTGAAG | | AAGAAGTTAGATGGCAGACA |
| *Aqp7* | GCTATCTCGGTGTCAACTT | | ACTTCTTCCAGGTCATTCG |
| *Plin1* | GACCTACAACAGCACCAA | | GGAACTCTACCACCTTCTC |
| *Plin2* | CTGGCTTCCTTGGTGATG | | AGTATGCTCCTTGTCTTAGTC |
| *Cpt2* | CAGCACAGCATCGTACCCA | | TCCCAATGCCGTTCTCAAAAT |
| *Fabp1* | GGAAGGACATCAAGGGGGTG | | TCACCTTCCAGCTTGACGAC |
| *Fabp4* | ACCGAGATTTCCTTCAAACT | | TAACACATTCCACCACCAG |
| *Acadm* | AGGGTTTAGTTTTGAGTTGACGG | | CCCCGCTTTTGTCATATTCCG |
| *Srebp1c* | GGAGCCATGGATTGCACATT | | GGCCCGGGAAGTCACTGT |
| *Pparγ* | AAGACCACTCGCATTCCT | | ACAGACTCGGCACTCAAT |
| *Fans* | CTCCTGAAGCCGAACACCTCTG | | ACCTTGCTCCTTGCTGCCATC |
| *Acc1* | GCAGACCACTATGTTCCA | | GCCTTCAGACCATCATCC |
| *Elovl6* | GAGAACGAAGCCATCCAA | | GCACCGAATATACTGAAGAC |
| *Cd36* | GGTCTATCTACGCTGTGTT | | GTATGTCCTATGCTCATCTTC |
| *Ldlr* | AGGCTGTGGGCTCCATAGG | | TGCGGTCCAGGGTCATCT |
| *Tnfα* | GCCACCACGCTCTTCTGTCTAC | | GACGGCAGAGAGGAGGTTGACT |
| *Il6* | GACCTGTCTATACCACTTCAC | | GTGCATCATCGTTGTTCATAC |
| *Il1β* | TGGACCTTCCAGGATGAGGACA | | GTTCATCTCGGAGCCTGTAGTG |
| *Ly6c* | GCAGTGCTACGAGTGCTATGG | | ACTGACGGGTCTTTAGTTTCCTT |
| *Gapdh* | CTCCCACTCTTCCACCTTCG | | GCCTCTCTTGCTCAGTGTCC |
| **Human primer sequences** | | | |
| **Gene name** | **Forward primer** | **Reverse primer** | |
| *GAPDH* | GGAGCGAGATCCCTCCAAAAT | GGCTGTTGTCATACTTCTCATGG | |
| *KIF13B* | TTGTGAATCTGAATGCTGAC | CTGGTGTTCTTCTGAGGAG | |

**Table S5 Body weight-adjusted ANCOVA-like analysis of raw indirect calorimetry parameters in *Kif13b^AKO^* mice**

| Outcome | Model | Genotype effect β | 95% CI | Genotype F value | Genotype P value | Body weight effect β | Body weight F value | Body weight P value | R² | Interpretation |
| --- | --- | --- | --- | --- | --- | --- | --- | --- | --- | --- |
| VO_2_ | Intercept + genotype + body weight | -4.260 | -24.06 to 15.54 | F(1,9) = 0.2369 | 0.6381 | 0.07827 | F(1,9) = 0.003721 | 0.9527 | 0.2034 | No significant genotype effect after body weight adjustment |
| VCO_2_ | Intercept + genotype + body weight | -4.260 | -24.06 to 15.54 | F(1,9) = 0.2369 | 0.6381 | 0.07827 | F(1,9) = 0.003721 | 0.9527 | 0.2034 | No significant genotype effect after body weight adjustment |
| EE | Intercept + genotype + body weight | -0.03117 | -0.1264 to 0.06404 | F(1,9) = 0.5484 | 0.4778 | 0.001504 | F(1,9) = 0.05938 | 0.8129 | 0.4490 | No significant genotype effect after body weight adjustment |

Raw VO_2_, VCO_2_ and energy expenditure (EE) values were analyzed using an ANCOVA-like multiple linear regression model in GraphPad Prism 10.0. Genotype was included as a categorical factor, and body weight was included as a covariate. The model included an intercept and the main effects of genotype and body weight. The control group was set as the reference level. β represents the adjusted effect of *Kif13b* deficiency compared with the control group at a given body weight.

**Table S6 Body weight-adjusted ANCOVA-like analysis of raw indirect calorimetry parameters in DIO mice**

| Outcome | Model | Genotype effect β | 95% CI | Genotype F value | Genotype P value | Body weight effect β | Body weight F value | Body weight P value | Interpretation |
| --- | --- | --- | --- | --- | --- | --- | --- | --- | --- |
| VO_2_ | Intercept + genotype + body weight | 13.23 | -6.124 to 32.58 | F(1,9) = 2.391 | 0.1565 | -0.8353 | F(1,9) = 0.6051 | 0.4566 | No significant genotype effect after body weight adjustment |
| VCO_2_ | Intercept + genotype + body weight | 12.65 | -2.626 to 27.92 | F(1,9) = 3.509 | 0.0938 | -0.6995 | F(1,9) = 0.6806 | 0.4307 | No significant genotype effect after body weight adjustment |
| EE | Intercept + genotype + body weight | 0.0675 | -0.02544 to 0.1604 | F(1,9) = 2.699 | 0.1348 | -0.00410 | F(1,9) = 0.6316 | 0.4472 | No significant genotype effect after body weight adjustment |

Raw VO_2_, VCO_2_ and energy expenditure (EE) values were analyzed using an ANCOVA-like multiple linear regression model in GraphPad Prism 10.0. Genotype was included as a categorical factor, and body weight was included as a covariate. The model included an intercept and the main effects of genotype and body weight. The control group was set as the reference level. β represents the adjusted effect of KIF13B knockdown compared with the control group at a given body weight.

**Table S7 Key resources table**

| REAGENT or RESOURCE | SOURCE | IDENTIFIER | |
| --- | --- | --- | --- |
| Antibodies | | | |
| Rabbit Polyclonal anti-GAPDH | Proteintech | 10494 | |
| Rabbit polyclonal anti-KIF13B | Sigma | SAB2101257 | |
| Rabbit polyclonal anti-CD68 | Boster | BM3639 | |
| Rabbit polyclonal anti-UCP1 | ABclonal | A5857 | |
| Rabbit polyclonal anti-LRP1 | MCE | HY-P80746 | |
| Rabbit polyclonal anti-PPARγ | Proteintech | 66936-1-Ig | |
| Donkey Anti-Rabbit IgG (Alexa Fluor® 555) | Abcam | AB150062 | |
| Goat Anti-Mouse IgG H&L (Alexa Fluor® 488) | Abcam | ab150113 | |
| Goat anti-rabbit IgG | ZSGB-BIO | ZB-2301 | |
| Goat anti-mouse IgG | ZSGB-BIO | ZB-2305 | |
| Virus strains | | | |
| AAV8-*shKif13b* | Genomeditech | N/A | |
| AAV8-*mcherry* | Genomeditech | N/A | |
| Biological samples | | | |
| Human adipose tissue samples | the Department of General Surgery at Peking University Third Hospital | Peking University Third Hospital Medical Science Research Ethics Committee (protocol No. M2024238) | |
| Chemicals, peptides, and recombinant proteins | | | |
| Insulin | NovoNordisk | NovolinRInsulinU100-10mL | |
| Oil red O | Sigma-Aldrich | O0625-100G | |
| RIPA | APPLYGEN | C1053-500 | |
| Phosphatase Inhibitor Cocktail | Roche | 4906845001 | |
| Protease Inhibitor Cocktail | Roche | 04693132001 | |
| DMEM/F12 | Gibco | 11320033 | |
| Bovine serum albumin | Sigma-Aldrich | A9056 | |
| DMEM | Gibco | 11965118 | |
| Penicillin-Streptomycin | Gibco | 15140122 | |
| Mounting Medium with DAPI | ZSGB-BIO | ZLI-9557 | |
| Goat serum | ZSGB-BIO | ZLI-9022 | |
| Diaminobenzidine (DAB) | ZSGB-BIO | ZLI-9018 | |
| Optimal cutting temperature (OCT) | Sakura | 4583 | |
| Trizol Reagent | Invitrogen | 15596018 | |
| Protein A/G agarose beads | Solarbio | M2400 | |
| Collagenase Type II | Gibco | 17101015 | |
| Red Blood Cell Lysis Buffer | Beyotime | C3702 | |
| Insulin | MCE | HY-P0035A | |
| 3-isobutyl-1-methylxantine | MCE | [HY-12318](https://www.medchemexpress.cn/IBMX.html) | |
| Dexamethasone | MCE | HY-14648 | |
| Rosiglitazone | MCE | HY-17386 | |
| PPARγ Luciferase Reporter Plasmid | Yeasen | 11511ES03 | |
| pGMR-TK Luciferase Reporter Plasmid | Yeasen | 11557ES03 | |
| Lipofectamine RNAiMAX | Invitrogen | 13778150 | |
| Liposomal Transfection Reagent | Yeasen | 40802ES03 | |
| D-(+)-Glucose | Sigma-Aldrich | G8270 | |
| Fetal bovine serum | TransGen Biotech | FS401-02 | |
| **Penicillin-streptomycin** | TransGen Biotech | FG101-01 | |
| Opti-MEM medium | Gibco | 31985070 | |
| Critical commercial assays | | | |
| Pierce BCA Protein Assay Kit | Thermo Fisher | 23225 | |
| Insulin ELISA kit | FUJIFILM Wako | 638-01489 | |
| Easy Pure Genomic DNA Kit | Transgene | EE101-02 | |
| Dual Luciferase Reporter Gene Assay Kit | Yeasen | 11402ES60 | |
| IL-6 ELISA kit | Ruixinbio | RXW203049M | |
| IL-1β ELISA kit | Ruixinbio | RXW203063M | |
| Triglyceride Assay Kit | BioSino | 100000220 | |
| Cholesterol Assay Kit | BioSino | 100000180 | |
| Glucose Assay Kit | BioSino | 100000240 | |
| LabAssay ™ NEFA Kit | FUJIFILM Wako | 633-52001 | |
| TransScript® First-Strand cDNA Synthesis SuperMix | TransGen Biotech | AT301-03 | |
| TransStart® Top Green qPCR SuperMix (+Dye II ) | TransGen Biotech | AQ132-24 | |
| Alanine aminotransferase (ALT) kit | Nanjing Jiancheng Bioengineering Institute | C009-2-1 | |
| Aspartate transaminase (AST) kit | Nanjing Jiancheng Bioengineering Institute | C010-2-1 | |
| C-C motif chemokine ligand 5 (CCL5) ELISA kit | Abcam | Ab100739 | |
| Nuclear protein extraction kit | R0050 | Solarbio | |
| Experimental Models: Organisms/strains | | | |
| C57BL/6J mice | Vital River Laboratories | | N/A |
| *ob/ob* mice | Huafukang Biotech | | N/A |
| *Kif13b*^AKO^ mice | This paper | | N/A |
| Software and algorithms | | | |
| GraphPadPrism | GraphPadSoftware,SanDiego,CA | | Version 10 |
| ImageJ | NIH | | N/A |
| Microsoft Excel | Microsoft | | Version16.93.1 |
| ImageScope | Leica | | N/A |
| ImagePro Plus | Media Cybernetics | | Version 6 |
| Other | | | |
| Chow diet (CD) | Beijing Keao–Xieli Feed Co., Ltd | | 1016706714625204224 |
| High-fat diet (HFD) | Research Diet | | D12492 |

**References**

1.	Miao GL, Zhang WX, Xu YT, et al. Motor protein KIF13B orchestrates hepatic metabolism to prevent metabolic dysfunction-associated fatty liver disease. *Mil Med Res.* 2025;12(1):11.

2.	Wolf FA, Angerer P, Theis FJ. SCANPY: large-scale single-cell gene expression data analysis. *Genome Biol.* 2018;19(1):15.

3.	Lopez R, Regier J, Cole MB, Jordan MI, Yosef N. Deep generative modeling for single-cell transcriptomics. *Nat Methods.* 2018;15(12):1053-1058.
